# BRCA Mutations—The Achilles Heel of Breast, Ovarian and Other Epithelial Cancers

**DOI:** 10.3390/ijms24054982

**Published:** 2023-03-05

**Authors:** Anna P. Loboda, Leonid S. Adonin, Svetlana D. Zvereva, Dmitri Y. Guschin, Tatyana V. Korneenko, Alexandra V. Telegina, Olga K. Kondratieva, Sofia E. Frolova, Nikolay B. Pestov, Nick A. Barlev

**Affiliations:** 1 Laboratory of Molecular Oncology, Phystech School of Biological and Medical Physics, Moscow Institute of Physics and Technology, 141701 Dolgoprudny, Russia; 2 Institute of Biomedical Chemistry, 119121 Moscow, Russia; 3 School of Medicine, Nazarbayev University, Astana 010000, Kazakhstan; 4 Group of Cross-Linking Enzymes, Shemyakin-Ovchinnikov Institute of Bioorganic Chemistry, 117997 Moscow, Russia; 5 Chumakov Federal Scientific Center for Research and Development of Immune-and-Biological Products, 108819 Moscow, Russia; 6 Institute of Cytology, Tikhoretsky ave 4, 194064 St-Petersburg, Russia

**Keywords:** breast cancer, ovarian cancer, PARP inhibitors, Alu repeats, protein-protein interactions

## Abstract

Two related tumor suppressor genes, *BRCA1* and *BRCA2*, attract a lot of attention from both fundamental and clinical points of view. Oncogenic hereditary mutations in these genes are firmly linked to the early onset of breast and ovarian cancers. However, the molecular mechanisms that drive extensive mutagenesis in these genes are not known. In this review, we hypothesize that one of the potential mechanisms behind this phenomenon can be mediated by Alu mobile genomic elements. Linking mutations in the *BRCA1* and *BRCA2* genes to the general mechanisms of genome stability and DNA repair is critical to ensure the rationalized choice of anti-cancer therapy. Accordingly, we review the literature available on the mechanisms of DNA damage repair where these proteins are involved, and how the inactivating mutations in these genes (BRCAness) can be exploited in anti-cancer therapy. We also discuss a hypothesis explaining why breast and ovarian epithelial tissues are preferentially susceptible to mutations in *BRCA* genes. Finally, we discuss prospective novel therapeutic approaches for treating BRCAness cancers.

## 1. Introduction

The first case of hereditary cancer was described in 1866 by Pierre Paul Broca, when he documented the development of breast and ovarian cancers within his wife’s family. It took almost 130 years to decipher the genetic mechanism behind this hereditary cancer syndrome. This was completed by Mary Claire-King and her colleagues, who published a linkage analysis of families with an early onset of breast cancer (BC) and identified the gene locus of *BRCA1* (BReast CAncer 1) at 17q21 [1]. The gene responsible for this phenotype was cloned in 1994. Shortly thereafter, the *BRCA2* gene was linked to chromosome 13, and cloned [2]. The products of these genes are functionally classified as tumor suppressors, meaning that inactivation of both copies of either gene is strongly associated with carcinogenesis. BRCA1 and BRCA2 proteins lack obvious structural homology, whereas a segment of BRCA1 is homologous to its partner, the BARD1 protein. In contrast to the canonical tumor suppressor inactivation mechanism, whereby one allele of a tumor suppressor gene is mutated and the other is either deleted or epigenetically inactivated (“loss of heterozygosity” principle, LOH), the *BRCA*-mutated cancerous cells frequently bear the remaining alleles in the wild-type state [3]. In this case, mutations in the *BRCA1* or *BRCA2* genes are often preceded by mutations in other critical tumor suppressor genes, *PTEN* and/or *TP53* [4]. Reversion of germline *BRCA* mutations in growing cancers is also common [5]. This indicates that haploinsufficiency may be the major basis for early development of BC in *BRCA1/2* pathogenic mutation carriers. Importantly, since the products of these genes are involved in the DNA damage response, the *BRCA* mutation status has profound significance for the selection of appropriate therapeutic interventions.

## 2. Epidemiology of Cancer and BRCA1,2 Mutations

### 2.1. Pan-Cancer Overview

Despite their close functional connection, *BRCA1* and *BRCA2* have somewhat different effects on cancer development and progression. For example, *BRCA1* and *BRCA2* each correlate with different subtypes of BC. *BRCA1* mutations are linked preferentially to the triple negative form of BC (estrogen receptor negative, progesterone receptor negative, and HER2 negative, TNBC), whereas *BRCA2*-associated breast cancers are generally estrogen receptor-positive [6], and phenotypically different (mostly luminal-like BC) [7]. Furthermore, mutations in *BRCA2* are more often associated with other types of epithelial cancer, including male BC, pancreatic cancer, and prostate cancer, than *BRCA1* mutations [8]. The expectancy for OC to occur in either of these genes in various tumors is also different. For example, for *BRCA1* mutations, the risk of OC is 40 to 45%, compared to 10–20% for *BRCA2,* as well as an earlier onset of OC for *BRCA1* cases [9].

Mutations in the *BRCA1* gene are mostly associated with hereditary cancers and are rarely found in sporadic cancers (compare more than 300 germline mutations for familial BC and/or OC, with only a few somatic ones in sporadic BC [10]). However, these rare cases are quite interesting, since they may result either from functional inactivation of *BRCA1* due to low gene expression, or from incorrect subcellular localization of the encoded protein [11].

Since *BRCA1* is a tumor suppressor, and is directly involved in the double-strand break (DSB) repair process, it is not surprising that the mutation status of this gene serves as a prediction marker for a high risk of carcinogenesis. Carriers of germline mutations in the *BRCA1* gene are prone to developing mostly BC and/or OC [12]. Although *BRCA* mutations are also found in many other types of tumors, they apparently do not have any detectable effect on cancer incidence in the brain, colon, bladder, kidneys, cervix, or lungs, nor an increased risk of melanoma [13,14]. However, *BRCA* mutation status often correlates with the severity of the disease and a shorter overall survival [15].

### 2.2. Ovarian Cancer

In total, 90% of ovarian cancers (OCs) are identified as epithelial OC (EOC), which is further subdivided according to histological characteristics into: low-grade serous; clear cell; endometrioid and mucinous [16]; and the most common, high-grade serous (HGSOC). The latter accounts for about 70% of all cases of EOC [17]. Importantly, approximately 15–20% of patients with HGSOC have germline mutations in *BRCA1* or *BRCA2* [18,19]. The presence of such *BRCA* mutations has also been reported in other histological subtypes of EOC [19,20].

Hereditary ovarian cancers are characteristic of three autosomal dominant familial syndromes: BC and/or OC, site-specific OC, and Lynch (hereditary non-polyposis colorectal cancer) syndrome [21]. Meanwhile, a familial history of OC, especially when associated with *BRCA1* mutations, poses a significant lifetime risk of developing the disease. Thus, 39–44% of women who inherit a *BRCA1* oncogenic-driving mutation develop OC by age 70–80 [22], and diagnosis at a later stage significantly worsens prognosis [23]. However, there is evidence that mutations in the *BRCA1* gene are associated with an increase in progression-free survival (PFS) [18,24,25,26]. This may be due to an increased sensitivity of such patients to treatment with platinum-containing drugs [12] or poly(ADP-ribose) polymerase (PARP) inhibitors.

Oncogenic mutations in *BRCA1* can be germline or somatic. According to the results of several independent studies, somatic mutations make up a significant part of all observed mutations in this gene among patients with OC [27,28,29,30]. Overall, somatic BRCA mutations occur in approximately 5–7% of OC cases [31]. The existence of somatic mutations fits into the concept of “BRCAness”, in which germline mutations of *BRCA1* or *BRCA2* are not detected, but the DNA repair defect occurs due to problems in the process of homologous recombination [32].

Studies have not revealed a significant difference in the course and aggressiveness of OC in patients with somatic or germline *BRCA1* mutations. Similarly to patients with congenital *BRCA1* mutations, patients with somatic *BRCA1* mutations showed an increased sensitivity to platinum-containing drugs and olaparib, a PARP inhibitor [29,33].

### 2.3. Breast Cancer

BC is one of the most common types of cancer diagnosed in women. This disease can also occur in men, although much less frequently. Molecular subtypes of BC include luminal A, luminal B, HER2-positive, triple negative, claudin-low, and normal-like, with other molecular markers important for classification being ERα+, PR, EGFR, CK5/6, VEGF, KI67, TNBC, MES, IM, and LAR [34]. Tumors associated with a *BRCA1* mutation are more likely to be triple-negative BC (TNBC), which is more aggressive and difficult to treat than other types [7,35].

BC caused by a mutation in the *BRCA1* gene has a higher rate of mitosis and greater lymphatic permeability than sporadic BC, as well as a higher frequency of somatic mutations in the p53 gene [34]. Women who inherit pathogenic *BRCA1* mutations face a very high lifetime risk of developing BC: 60% to 80% by the age of 80 years [34,36]. Two-thirds of the *BRCA1* mutations found in BC are germline, and the remaining third relates to somatic mutations [37,38,39]. Germline and somatic *BRCA1* mutations are currently assumed to be biologically equivalent [40]. There is evidence that tumors carrying *BRCA1* germline mutations have biological signatures similar to tumors with somatic *BRCA1* mutations [41,42]. However, there is also data showing that somatic mutations of the *BRCA1* gene have not been identified in BC without concurrent germline mutations [43], which may explain the small difference between tumors with somatic and germline *BRCA1* mutations.

### 2.4. Pancreatic Cancer

Pancreatic cancer is reported to be the third most common cancer associated with *BRCA* mutations [44]. A family history of pancreatic cancer is found in 5–10% of patients with pancreatic cancer. Pancreatic ductal adenocarcinoma (PDAC) occurs especially frequently in families with OC or BC [45]. Pathogenic mutations in *BRCA2* occur in 2% of patients with pancreatic cancer, and mutations in *BRCA1* in 1% of patients. Approximately 7% of patients with pancreatic cancer may carry germline mutations in *BRCA1*/2. In patients with hereditary pancreatic cancer, the prevalence of *BRCA1*/2 mutation carriers is estimated at 4.9–26%. Mutations in *BRCA2* appear to be more common in pancreatic cancer. Furthermore, these mutations are considered to be more dangerous and increase the risk of developing pancreatic cancer severalfold [46].

### 2.5. Prostate Cancer

Mutations in the *BRCA1* and *BRCA2* genes increase the risk of developing prostate cancer. Some results indicate significantly lower survival rates and a more aggressive course of the disease [47,48]. Male carriers of a *BRCA2* gene mutation have a significantly increased risk of developing prostate cancer [49].

### 2.6. Mutations and the Founder Effect

**Figure 1:**
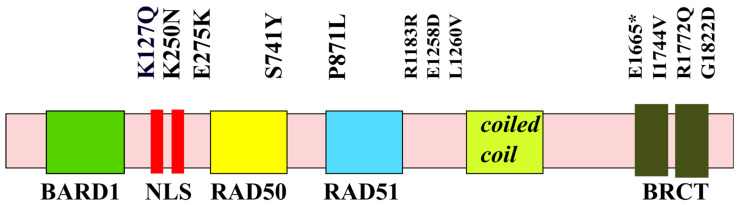
Distribution of major pathogenic mutations along the structural and functional domains of *BRCA1*. * indicates a nonsense mutation.

Pathogenic mutations in *BRCA1*/2 have been found throughout the coding region of this gene and at splicing sites (Figure 1). Most mutations in both genes are small insertions or deletions resulting in frameshifts, nonsense mutations, or splice-site changes that cause the stop codon to occur prematurely [21]. Therefore, it is quite difficult to isolate the regions that are most susceptible to the deleterious mutations common among various types of cancer.

In respect to BC, there are studies highlighting exon 10 (usually termed exon 11 for historical reasons) of *BRCA1* as the most mutated in BC patients [34,50]. According to The Breast Cancer Information Core (BIC), a catalog of *BRCA1* and *BRCA2* mutations identified worldwide, the most commonly identified *BRCA1* mutations are 185delAG (16.5%), 5382insC (8.8%), and the C61G missense mutation (1.8%). However, exon 10 is longer than all other exons combined, thus physically providing more opportunities for mutations to occur.

Additionally of note, there is a remarkable variation in the distribution of *BRCA1* mutations around the world; for example, some *BRCA1* variants are limited to geographically isolated regions or specific populations. This phenomenon is described as the “founder effect”. It has a profound influence on fundamental studies, diagnoses, and treatment approaches of *BRCA1*-associated cancers [51].

In some countries and ethnic communities, the spectrum of *BRCA1* mutations is strictly limited to a few founder mutations. For example, the founder effect in the population of Ashkenazi Jews is well described: three mutations in the *BRCA1* gene (*BRCA1* c.68_69delAG, c.5266dupC and *BRCA2* c.5946delT) account for 98–99% of the identified mutations, and are found in approximately 2.6% of the Ashkenazi Jewish population [52].

In Russia, the most common *BRCA1* mutation is c.5266dupC, accounting for about 90% of all *BRCA1* mutations. Other less common mutations found in Western Russia are c.4035delA, c.181T > G and c.68_69delAG [53,54,55].

## 3. Molecular Evolution of BRCA and Links to Human Cancers

Evolutionarily, both *BRCA1* and *BRCA2* are ancient genes that are indispensable for high-fidelity DSB DNA repair in most of *Eukaryota*. However, it should be mentioned that *BRCA1* seems to be absent from all fungi, and *BRCA2* was not found in yeast. However, the carboxyl-terminal BRCT domain (Figure 1) homologs were identified in several yeast proteins (e.g., Rad4 and Rad9), indicating that the function of BRCA1 and BRCA2 can be distributed between several yeast proteins involved in the process of DNA repair [56,57].

Since the harmful effects of mutations in the BRCA genes are developed only later in life, these mutations are likely to be passed on to future generations. Because these mutations do not affect reproductive fitness, the purging force of natural selection will be weak and insufficient for consistently eliminating these mutations [58]. Therefore, mutations in *BRCA1* and *BRCA2,* especially because they are inherited in a dominant manner, may be considered as good illustrations of the mutation accumulation theory. In this situation, the dominant nature of *BRCA1*/2 mutations may decrease the fertility of female carriers through an accelerated depletion of ovarian reserve, as described in several independent reports (for example, [59,60]). Although the onset of menopause is largely unaffected [60], and hence the magnitude of this effect may be overestimated [61], it is worth mentioning that even a small decrease in age-associated fertility may have drastic consequences on the evolutionary scale.

It is assumed that *BRCA1* or *BRCA2* mutations promote carcinogenesis predominantly in breast and ovarian epithelia because, since menstrual cycles periodically create a hormone-dependent enrichment in the female hormone-responsive tissues of reactive oxygen species (ROS), there would be a demand for an augmented expression of the genes responsible for antioxidant defense and DNA repair machinery against genotoxic metabolites including, for example, endogenous quinones derived from 2- and 4-hydroxyestradiols [62]. This may be a plausible explanation for the fact that mostly female hormone-responsive tissues are exquisitely sensitive to germline mutations in the *BRCA1* and *BRCA2* genes [63]. It should be noted, however, that this highly tempting hypothesis of tissue-specific carcinogenesis cannot account for the increase in pancreatic and prostate cancer incidences (albeit to much lower levels compared to breast and ovarian tissues). Indeed, the problem of tissue-specificity of oncogenic effects exerted by ubiquitously expressed genes is rather multifactorial and requires additional studies [64].

## 4. A Potential Mechanism for Enrichment of Mutations in the BRCA Genes

Here, we attempt to highlight the importance of the intrinsic genetic mechanisms that control genomic instability in humans, specifically Alu repeat elements. They occupy almost 11% of the human genome and exert wide-ranging influences on gene expression. Alu elements are ~300 base pair retrotransposon sequences that are normally silenced by DNA methylation and heterochromatin formation. However, in the germline, Alu elements are more active and may significantly contribute to genetic diseases and population diversity. In particular, we argue that Alu repeats may significantly contribute to the mutagenesis of *BRCA1*/2 genes through several mechanisms: direct insertional mutagenesis and/or as an abundant source of repetitive sequences that, in turn, contribute to non-allelic homologous recombination, which would result in genetic deletions and duplications [65].

Over the last 20 years, research has expanded to improve the understanding of BRCA-related BC and OC, specifically in how they respond to treatment, as well as the expected clinical course. Better characterization of alterations in these genes may enable the development of new, targeted therapies, or broaden the clinical application of current therapies [12].

### A Hypothetical Role of Transposable Elements (TEs) in BRCA-Associated Carcinogenesis

**Figure 2:**
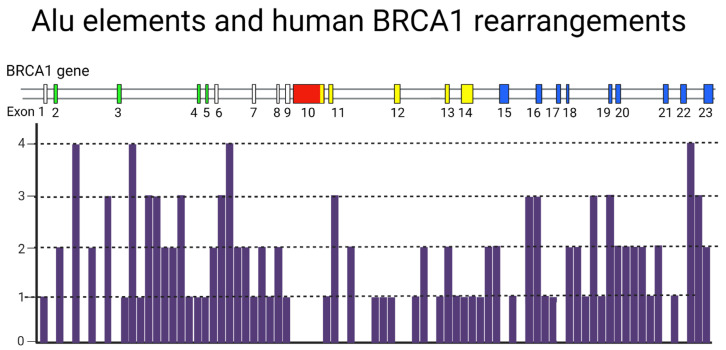
Distribution of Alu repeats in the human *BRCA1* gene. Exon colors correspond to functional domains: green—RING; red—NLS-containing domain; yellow—coiled coil; blue—BRCT tandem.

*BRCA1*/*BRCA2* genes harbor a very high density of repetitive DNA elements that contribute to genetic instability [66]; the *BRCA1* gene contains 138 individual Alu elements [67], which occupy about 42% of intronic sequences (Figure 2). In addition, this gene includes 5% of various other repeats [68,69]. *BRCA2* contains almost 47% repetitive DNA elements, but only 17–20% of them are Alu repeats. These genes show a high probability of mutations that are associated primarily with Alu-mediated genomic rearrangements [70,71]. These rearrangements are more frequent in *BRCA1* than in *BRCA2*, probably due to the large number of Alu repeats in the gene sequence [72,73,74].

Although most genomic rearrangements were proven experimentally to be pathogenic by causing frameshifts and premature termination codons, some rearrangements have more ambiguous effects. In particular, this concerns in-frame deletions of redundant exons [75] or, conversely, some duplications, where additional copies of exons might be well tolerated by the organism without deleterious effects [76].

Almost 10% of BC cases are related to defects in the *BRCA1* or *BRCA2* genes [77]. Women with a familial history of confirmed *BRCA1* or *BRCA2* defects have been shown to possess a remarkably high lifetime risk of developing BC (80% and 60%, respectively) [78,79]. It was also shown that large rearrangements in *BRCA1,* but not *BRCA2*, play a notable role in the predisposition to breast and ovarian cancers in high-risk families of German origin [80]. Researchers analyzed 226 patients with a high-risk of hereditary BC and OC and described six large genomic alterations in the *BRCA1* locus. *BRCA1* mutations include a deletion of exon 5, a deletion comprising exons 5–7, a deletion of exons 1A, 1B, and 2, two duplications of exon 13, and a deletion of exon 17. However, nothing similar was found in the *BRCA2* gene. In another study, two families with a high risk of hereditary BC and OC were found to carry a 7.1 kb germline deletion, which includes exons 8 and 9. This deletion leads to a frameshift at the mRNA level [81].

Only a few other studies have investigated *BRCA2* rearrangements [82]. To date, about 16 cases of *BRCA2* germline rearrangements have been reported. It was shown that large genomic *BRCA2* rearrangements are observed in males in affected hereditary BC families, predominantly [83]. Genomic rearrangements of the *BRCA2* gene were present in 25 families, among which there was at least one man with BC. However, no *BRCA2* gene rearrangements were found in 114 families among women with BC [84]. These results raise the question of the possible existence of sex-related mechanisms of the gene rearrangements in the *BRCA2* gene.

The Alu-indirect insertion in exon 3 of *BRCA2*-c.156_157insAlu- is quite common in families with an inherited predisposition to BC and/or OC. Researchers found this mutation in 14 families (out of 208 tested) and it accounts for about a quarter of all mutations in the *BRCA1*/2 loci [85].

Thus, Alu-mediated rearrangements in the *BRCA1* and *BRCA2* genes, including deletions and insertions that lead to global genomic rearrangements of these genes, are closely associated with the predisposition to BC and OC.

## 5. Structure-Function Analysis of Human *BRCA1*

**Figure 3:**
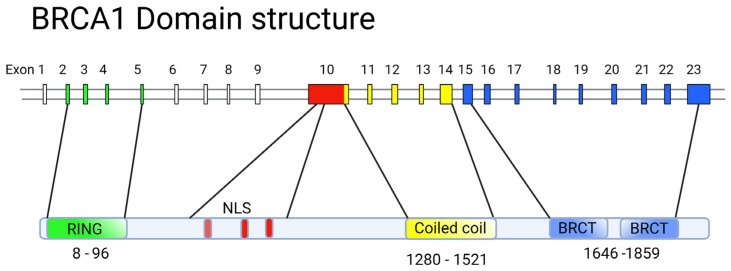
Alignment of the exon–intron structure of *BRCA1* and the domain composition of the corresponding protein. Exon colors correspond to functional domains. Note that exon 10 is frequently referred to as exon 11 for historical reasons.

The BRCA1 protein is involved in vital processes in the nucleus, namely transcription, DNA repair (including the repair of transcription-related DNA damage), and cell cycle control. Accordingly, BRCA1 is localized to discrete sub-nuclear structures associated with DNA replication or repair. DNA damage induces BRCA1 phosphorylation and recruitment to specific foci containing DNA repair proteins, where BRCA1 is deemed to act as a scaffold for the assembly of various multiprotein complexes. Despite the large molecular weight of BRCA1 (1863 amino acid residues [86]), only two conserved domains can be distinguished in its structure: the N-terminal RING domain (exons 2–6) [87] that encompasses 100 amino acid residues; and two tandem C-terminal BRCT domains, with 90 amino acid residues each [88], encoded by the end of exon 16, and exons 21–24, respectively (Figure 3). The region of the protein located between these two terminal domains is structurally variable between mammalian BRCA1 homologues. It is believed to be intrinsically disordered, yet it is critical for the proper functioning of BRCA1, along with the other two conserved domains (Figure 3).

### 5.1. The RING Domain

**Figure 4:**
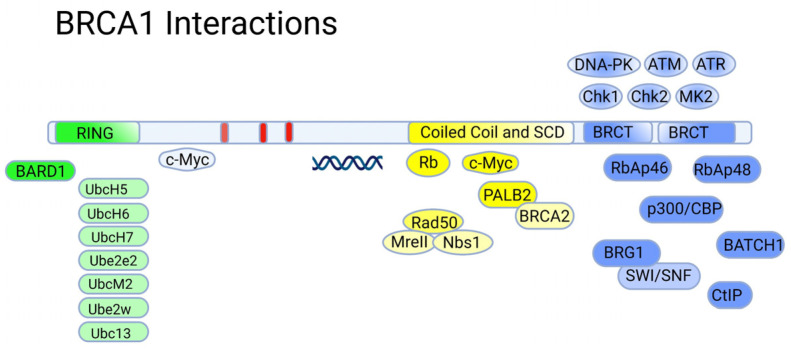
Major protein interactions aligned with regions of BRCA1.

The DNA-binding RING (Really Interesting New Gene) domain has an E3 ubiquitin ligase activity, being a scaffold for the interaction with the corresponding E2 ubiquitin ligases such as UbcH5, UbcH6, UbcH7, Ube2e2, UbcM2, Ube2w, and Ubc13 (Figure 4) [89]). The ubiquitin ligase activity of BRCA1 is stimulated by the formation of a heterodimer with the BARD1 protein [90]. The latter also contains a RING domain and tandem BRCT domains, and shares some structural similarity to BRCA1 [91]. Like BRCA1, BARD1 tends to form specific foci in the nucleus in S-phase of the cell cycle that overlap with the ones formed by BRCA1, suggesting that the formation of the BRCA1/BARD1 complex is cell-cycle-dependent [92].

**Figure 5:**
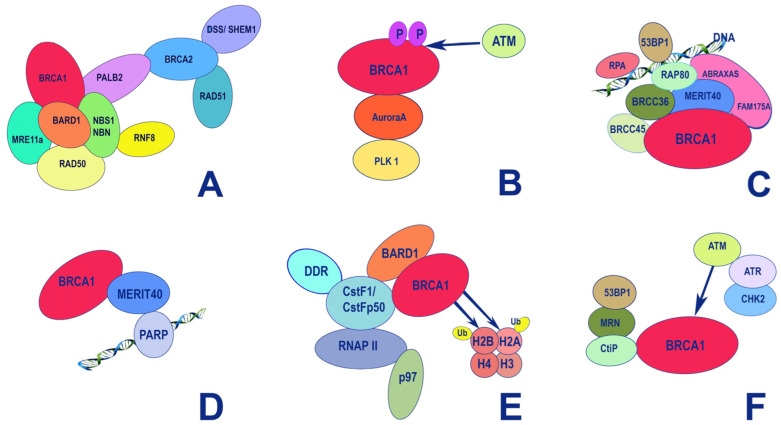
Major functional protein–protein interactions of *BRCA1* and *BRCA2* and the functional roles of these complexes. (**A**) DNA homologous recombination; (**B**) G2/M transition after recovery from DNA damage; (**C**) DNA damage resistance, and G2-M checkpoint control; (**D**) regulation of immediate cellular response to single-strand DNA damage; (**E**) histone ubiquitination concurrent with transcription regulation and DNA repair; (**F**) response to double-strand DNA damage.

The formation of a complex with BARD1 is necessary for the stabilization of BRCA1 at the protein level. Furthermore, this interaction is apparently important for the nuclear localization of BRCA1. The BRCA1/BARD1 heterodimers are involved in the DNA repair of double-strand breaks, and hence the preservation of DNA integrity, including the process of resolving impaired replication forks (for more details, see [89]). Mechanistically, the BRCA1/BARD1 complex is recruited by the RAP80 protein to sites of DNA damage [93], where the BRCA1/BARD1 ubiquitin ligase is employed to modulate the activity of DNA damage response factors (Figure 5). Additionally, the proteolytic activity of 26S proteasomes is also modulated by DNA damage stimuli, thereby adding another level of complexity to the regulatory mechanisms of DNA repair [94].

Importantly, the BRCA1/BARD1 heterodimers also interact with the RNA polymerase II holoenzyme. However, BRCA1 does not show an increased affinity for specific DNA sequences, except for some abnormal structures (branched DNA formations) [95]. This does not allow BRCA1 to be considered a bona fide transcription factor. Considering the fact that in the central unstructured and C-terminal regions of BRCA1, there are many binding sites for various transcription factors, chromatin remodeling factors, and DNA-damage response factors, it would be fair to say that BRCA1, in complex with BARD1, forms a scaffold for the surveillance of genome integrity control during transcription [96]. However, there are also cases when BRCA1 acts as a corepressor; for example, the transcription factor ZBRK1 suppresses the transcription of its target genes in a BRCA1/CtIP-dependent manner [97]. ZBRK1 acts as a metastatic suppressor by directly regulating MMP9 in cervical cancer.

### 5.2. The BRCT Domain

The C-terminal region of BRCA1 (1650–1863) is occupied by two BRCT (BRCA1-C-Terminal) tandem-repeat domains connected by a 22-amino-acid linker [98]. The BRCT domains are protein-binding modules that recognize the phosphorylated motif pSer-x-x-Phe [99]. Due to this, BRCA1 is included in the signaling cascades triggered by DNA damage as a scaffolding platform for the interactions of various kinases and other proteins involved in the regulation of the cell cycle [100]. Additionally, BRCA1 itself undergoes reversible phosphorylation upon DNA damage [101] by key regulators of the DNA damage response: PIKK kinases (ATM, ATR, and DNA-PK) [102] and checkpoint effector kinases (Chk1, Chk2 and MK2) [103]. Phosphorylation of BRCA1 also creates new sites for complex protein–protein interactions affecting various aspects of DNA damage and repair (Figure 5).

**Figure 6:**
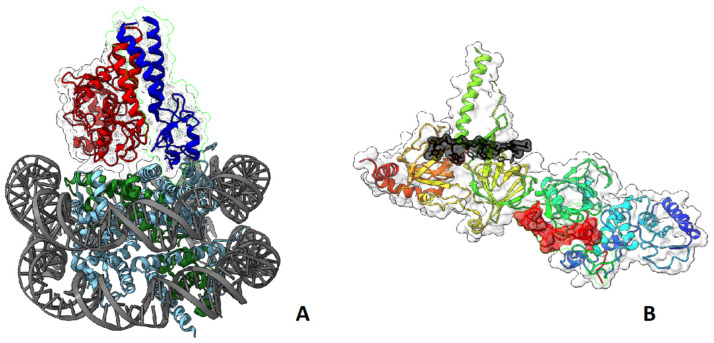
(**A**) Three-dimensional structure of the nucleosome in complex with BRCA1 and BARD1 fragments (BRCA1—red, BARD1—blue, histone 2A—green) [104]; (**B**) structure of BRCA2 with SEM1/DSS1 and single stranded DNA (SEM1/DSS1—red, ssDNA—black) [107].

The BRCA1/BARD1 complex senses the ubiquitination status of histone H2A and works as a ubiquitin ligase of this histone. These activities play important roles in the choice between HR or NHEJ during DNA damage repair: BRCA1 acts as a mediator for HR, antagonizing the 53BP1-mediated NHEJ pathway [104,105,106] (Figure 6A). BRCA2, complexed with SEM1/DSS1 and ssDNA [107] (Figure 6B), functionally interacts with recombinase RAD51, PALB2, ssDNA-specific endonuclease XPG/ERCC5, and BRCA1 [108].

### 5.3. BRCA1 and p53

*TP53 (p53*) is arguably one of the most significant tumor suppressor genes in humans. It is frequently mutated and several point mutations in its DNA-binding domain convert the p53 protein into an oncogene. That *TP53* mutations occur in tumors bearing *BRCA1* mutations suggests that the two genes may function in different signaling pathways to suppress tumorigenesis [109]. However, results from experiments in mice have shown that tumorigenesis occurs much more efficiently when both *BRCA1* and *TP53* are deleted, compared to *BRCA1* deletion alone [110], indicating that p53 is located downstream of BRCA1 in the same signaling pathway. Accordingly, mutations in *BRCA1* preceding mutations in the *TP53* gene, as seen in cases of familial BC, are not sufficient for tumor progression. Since *BRCA1*-null cells display genomic instability, it is likely that persistent intrinsic DNA damage in the presence of wild-type p53 leads to the extermination of such cells via p53-dependent cell cycle arrest and apoptosis.

There is another important fact that functionally links p53 and BRCA1: in response to various types of DNA damage, both p53 and BRCA1 become phosphorylated by DDR-dependent kinases, ATM and Chek1. Upon DNA damage, BRCA1 also interacts with another kinase, c-Abl [111]. The C-terminus of BRCA1 is phosphorylated by c-Abl in vitro. In vivo, BRCA1 is phosphorylated at tyrosine residues depending on ATM and irradiation. However, the tyrosine phosphorylation of BRCA1 does not disrupt the interaction between BRCA1 and c-Abl. Notably, cells with *BRCA1* mutations exhibit constitutively high c-Abl kinase activity, which does not increase when cells are exposed to gamma radiation. Probably, *BRCA1* mutations, due to defects in DNA repair, induce the kinase activity of c-Abl towards p53, which culminates in p53-dependent cell cycle arrest and cell death. In addition to phosphorylation and the subsequent activation of p53 transcriptional activity, c-Abl also stabilizes p53 on the protein level by inactivating its major inhibitor, E3 ligase Mdm2 [112]. Curiously, c-Abl also phosphorylates another tyrosine kinase, BTK [113]. In this respect, we have recently shown that BTK can phosphorylate p53, leading to its stabilization and transcriptional activation [114], suggesting a novel role for BTK as a potential tumor suppressor [115].

It is also known that BRCA1 and p53 are able to interact physically. Deletion analysis in the BRCA1 gene allowed for the identification of p53-interacting domains in the coiled–coiled region and in the second BRCT domain. On the other end, p53 interacts with BRCA1 at the C-terminus. BRCA1-mediated stabilization of the wild-type p53 protein occurs through upregulation of the p14ARF gene product, which in turn upregulates mouse p53 phosphorylation at serine 18 (equivalent to human serine 15). Exon 10 (historically exon 11) of *BRCA1* appears to be responsible for this, since cells with deletions of exon 10 in *BRCA1* are defective in p53 stabilization after DNA damage [116].

Functionally, this interaction converts BRCA1 into a p53 coactivator [117]. Perhaps not surprisingly, both proteins, p53 and BRCA1, transcriptionally regulate the expression of the *GADD45* gene, which induces growth arrest and DNA damage repair. Both *BRCA1*-deficient and *GADD45*-deficient cells displayed a G2/M cell cycle checkpoint defect and increased genome instability [118].

Collectively, these results suggest that the phenotypic manifestation of *BRCA1* tumorigenic mutations heavily relies on the spectrum of inactivation in other critical tumor suppressors, e.g., p53.

### 5.4. BRCA1 and BRCA2—A Summary on Normal Functions in Healthy Tissues

Both *BRCA1* and *BRCA2* are ubiquitously expressed in human tissues and serve as important parts of the complex machinery that guards DNA integrity. Especially as demonstrated by gene knockout mice (reviewed in [119]), the complete absence of these genes is incompatible with normal development. However, many questions remain to be answered, such as the mutational rates in the germline on the evolutionary scale in different populations and species, especially with respect to the relatively fast evolution of *BRCA1* and *BRCA2* themselves, and especially in their unusually long central exons.

## 6. Survival of BRCA-Mutated Cancer Cells: Role of Tissue Microenvironment

One may wonder why the tumorigenic role of *BRCA1*/2 mutations is exemplified preferentially in BC cells, and not so much in other epithelial tissues. In this respect, it should be noted that mutated BC cells are largely derived from luminal progenitor cells. Although germline *BRCA1*/2 mutations occur stochastically in many tissues [120], the breast tissues of patients with oncogenic germline *BRCA1*/2 mutations have distinct histological features [121]. Premalignant lesions in this tissue also have certain molecular hallmarks, such as upregulated expression of progesterone receptor A [122].

### Hypothesis: Role of Breast Adipocytes in Early Progression of BRCA1/2 Mutated Microtumors

The survival of early malignant cells in the surrounding normal tissue is dependent on many factors, including escape from immune surveillance by natural killers. Indeed, it is physiological for the breast ductal epithelium to invade into adipose tissue and partially displace it during lactation [123]. Thus, breast adipocytes may sense the invasion of micro-metastatic or circulating breast tumor cells as a normal process, which would prevent inflammatory signaling in these niches.

In general, the role of adipocytes in cancer progression was highlighted in several excellent reviews [124,125,126,127,128]. It was suggested that adipocytes enhance cancer growth through the secretion of exosomes that contain tumor-promoting factors, e.g., TSP5 [129]. In this respect, BC-associated adipocytes may stimulate the onset of epithelial-mesenchymal transition (EMT) in BC cells by expressing exosomal TSP5 [124,130]. Mechanistically, breast adipocytes protect early breast tumor cells from ferroptosis and other ROS-mediated forms of cell death through the secretion of fatty acids [131], and the cross-talk between adipocytes and malignant cells may occur via secretion of leukemia inhibitory factor (LIF) and C-X-C subfamily chemokines in a positive feedback mode [132]. Also, these cancer-associated adipocytes undergo “browning”: the process of increasing the number of mitochondria [133]. This occurs concomitantly with inflammation-like signaling [134], and the stimulation of vascularization [135]. Collectively, breast adipocytes may create a unique natural tumor niche for BC cells with germline mutations in *BRCA1/2* genes. Furthermore, BC cells readily invade multiple tissues, such as the lungs, liver, bones, etc. Again, adipocytes may play an important role in allowing invading cells to colonize and proliferate [136].

## 7. Vulnerabilities of BRCA-Mutated Cancer Cells

However aggressive the *BRCA* mutant cancers may be, these mutations also give fast-growing cells certain features that may result in paradoxically better sensitivity to some cytostatic and targeted therapies. Indeed, patients with TNBC now have better prognoses if they bear the pathogenic *BRCA* mutations [6].

### 7.1. Platinum Complexes

Both platinum-containing drugs and PARP inhibitors (PARPi) are used to treat homologous recombination-deficient (HRD) cancers that have mutations in genes involved in double-strand DNA repair [137]. Platinum salts create DNA interstrand crosslinks that are extremely difficult to cleave in the absence of homologous recombination (HR), which leads to the death of HRD cancer cells [138]. Enhanced sensitivity of *BRCA1*/2-mutated cancers to platinum salts has been well documented in numerous studies, for instance, those on OC [139], pancreatic cancer [140], and BC [141]. If the normal copy of *BRCA1* or *BRCA2* is retained, the efficacy of platinum-based therapies is decreased [142]. Additionally, platinum resistance may develop upon reverse mutations in *BRCA1* [143].

### 7.2. PARP Inhibitors

**Figure 7:**
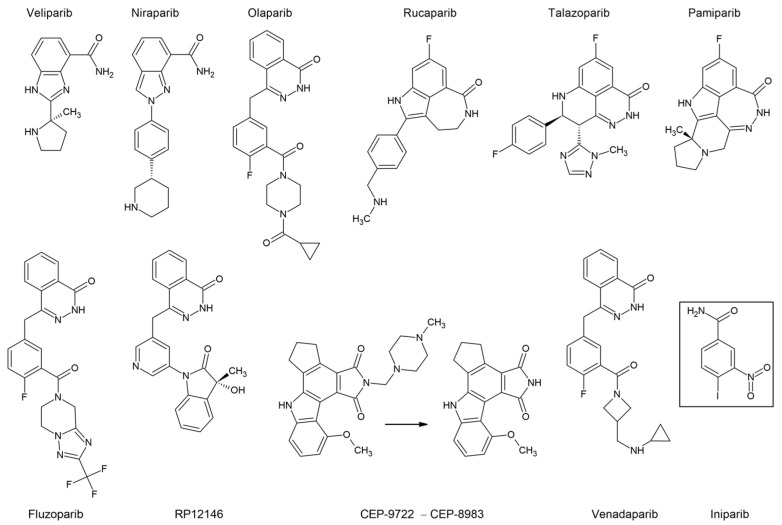
Structural formulae of PARP inhibitors. Upper row: PARP inhibitors already approved for clinical use. Lower row: some new prospective compounds with sponsored trials. The arrow indicates prodrug conversion. The box indicates unsatisfactory clinical data.

The exact mechanism of action for PARP inhibitors (Figure 7) has not yet been fully understood. Initially, they were developed as dissipaters of DNA repair and potent sensitizers of cancer cells to chemotherapy, but they also showed a significant independent effect on patients with mutations in the HR genes, primarily *BRCA1*. The effect of synthetic lethality for PARP inhibitors was shown in cells with the loss-of-function mutations in *BRCA1* [144]. There are several hypotheses about the mechanism of their combined action [137]. The main model posits that inhibitors bind to the PARP catalytic site, preventing its autoPARylation and further dissociation from the DNA. The latter ultimately leads to the collapse of the replication fork and DNA double-strand breaks that cannot be repaired by HR in cancer cells [145]. The increased sensitivity to these drugs in tumor cells with either somatic or germline *BRCA1* mutations suggests that the mechanism of HRD does not depend on whether the *BRCA1* mutation was inherited, or arose during the life of the patient.

Significant disturbances in the mechanism of DSB DNA repair in the absence of fully functional BRCA1 or BRCA2 make cancer cells particularly sensitive to PARP inhibitors, especially in the case of LOH. In this case, the same molecular features that make these cancers more aggressive also give them vulnerabilities that may be therapeutically exploited. There have been reports on the rather encouraging success of PARP inhibitors, even against relapsed *BRCA*-mutated cancers [146].

However, in the treatment of certain types of tumors, such as *BRCA1*/2-mutated and HER2-positive BC, the efficacy of talazoparib, a potent PARP1/2 inhibitor, did not surpass conventional chemotherapy [147]. This indicates that further personalization of anti-cancer therapy may improve the effectiveness of PARP inhibitors, as well as reduce their unwarranted use.

### 7.3. Boosting Synthetic Lethality by Drug Combinations

Currently, there is a number of ongoing clinical trials with patients recruited based on their *BRCA1*/2 status (Table 1 has been excerpted from Supplementary Table S1 to give a snapshot of the modern approaches being utilized to employ co-targeting beyond standard cytostatic regimens). However, future possibilities for specific new therapies are much wider. For example, the ubiquitination activity of *BRCA1* may become a prospective target for new synthetic lethality drugs [148]. PARP inhibition may be synergistically accompanied by blocking the RAD52 pathway of HR [149]. PARP inhibitors may be converted to more complex molecules with a double-specificity mechanism of action [150]. The action of olaparib and other PARP inhibitors may sometimes be enhanced by some unexpected supplements, such as antioxidants [151]. Combining the inhibition of PARP with the blocking of ATR by ceralesertib may potentially augment the anti-cancer effect of already-existing PARPi [152]. Further, DNA G-quadruplex binders such as pidnarulex may act in a similar manner, thus increasing the arsenal of drugs for *BRCA*-mutated cancers [153].

## 8. Future Perspectives

Over the past few decades, the clinical significance of *BRCA* mutations for the rational choice of anti-cancer therapy has been firmly established. In this respect, the synthetic lethal interaction between PARPi and *BRCA* mutations gives a remarkably successful example of how a fundamental discovery in molecular medicine can be translated into clinical cancer therapy. However, the next step of the problem is the multifariousness of PARPi resistance mechanisms (recently reviewed in depth by Jackson and Moldavan [154]) that eventually arise in patients with *BRCA* mutations in response to this therapy. In particular, Alu mobile elements regulate the expression of many genes, including the ones that mediate DNA repair [155]. This observation poses an interesting question of whether Alu repeats can be involved in the DNA damage repair process and serve as a potential mechanism for PARPi resistance in *BRCA* mutant cells [156]. Furthermore, the recently published data of the clinical trial of RITA suggest that patients treated with a PARPi, niraparib, displayed significantly longer PFS, compared to the placebo cohort, regardless of the presence or absence of intact HR repair [157]. This result indicates that PARPi might kill cancer cells in ways other than by affecting DNA repair, although the most feasible explanation is the inhibited PARylation of HR-participating proteins, including BRCA1 [158,159].

Theoretically, it can be hypothesized that a loss of BRCA by cancer cells should increase their susceptibility to various novel regimens of anti-cancer therapies due to the attenuated DNA repair. For example, therapeutic viral intervention seems to be a plausible therapeutic approach to treating BRCAness cancers, especially in combination with PARPi drugs [160]. However, it should be noted that PARP inhibition may activate genes linked to the normal interferon response in BRCA1,2-deficient cells [161] and this may explain the molecular basis of interference between the treatment with oncolytic viruses and PARPi. Therefore, one should pay attention to the *BRCA* mutational status when implementing new oncolytic viruses against BC and/or OC.

Managing *BRCA1* and *BRCA2* pathogenic mutations may include many options other than extensive testing and preventive surgery for such patients. The idea of long-term therapeutic interventions, such as hormone replacement, has long been discussed, but poses serious risks of adverse effects [162]. This concept is now re-emerging (discussed in [163]), due to the implementation of drug repurposing (Denosumab, Metformin, Letrozole, etc.; see Supplementary Table S1), as well as principally new approaches, such as adiponectin receptor-targeting molecules [164].

The p53 tumor suppressor plays an important role in inhibiting cancer progression, especially in response to chemotherapy or targeted therapy. Genomic inactivation of *TP53* by missense or nonsense mutations often leads to drug resistance in cancer cells. It was previously thought that, since wild-type p53 transcriptionally induces the expression of genes involved in DNA repair [165], then *TP53*-mutant cells with attenuated DNA repair would be more sensitive to PARP inhibitors which block homologous DNA repair. Accordingly, a deficiency of or mutations in the *TP53* gene have been shown to enhance the cytotoxicity of PARP inhibition in various tumors with mutations in *BRCA1*/2 [166]. However, recent studies in colorectal cancer have shown that, contrary to previous findings, wild-type p53 activity appears to be important for a full cytotoxic response to PARP inhibition [167], as PARP inhibitors have been found to activate the p53 pathway [168]. One of the explanations for this phenomenon may be the fact that it is wild-type, and not mutant, p53 that promotes the export of *BRCA1* from the nucleus, increasing the cellular deficiency of homologous repair [169]. Another explanation could be that *TP53* encodes a large number of microRNAs that target genes responsible for the repair of double- and single-stranded DNA breaks [170,171], thereby increasing the sensitivity of cancer cells to PARP inhibitors.

In this regard, the question arises of whether the combination of PARP and activators of p53 may have a synergistic effect. Since Mdm2 is the principal p53-specific E3 ligase that degrades p53 [172], it will be interesting to see whether inhibitors of the p53–Mdm2 interaction can be combined with PARP inhibitors. A number of new Mdm2 inhibitors are currently undergoing clinical trials [173]. Notably, we and our colleagues have also discovered several new inhibitors of p53 interaction with Mdm2, and these molecules exhibited strong apoptotic effects [174,175,176]. Future experiments will show whether the combination of p53 activators and PARP inhibitors is a viable therapeutic approach to treating BRCAness cancers.

Complex combinations, as expected, should be more effective, although more difficult and time-consuming to develop and adjust to practical regimens. For example, a combination of cisplatin, mitomycin C, and doxorubicin was reported to be more efficient than the respective double combinations [177]. Finally, there are multiple ways to boost standard neoadjuvant regimens, such as the addition of bevacizumab to anthracycline and taxane for patients with *BRCA1,2* mutations [178].

## 9. Conclusions

Further progress in fundamental studies on DNA repair, and the development of even more potent and specific drugs, may wield power over the intrinsic weaknesses of many cancers. Even relatively simple improvements in molecular diagnostics, such as the detection of cases with loss-of-function *BRCA1,2* mutations, may yield a highly positive impact on the therapeutic treatments for many oncological patients worldwide.

## Figures and Tables

**Table 1:** Representative ongoing clinical trials of drug combinations (PARPi and targeted therapies) against cancers with *BRCA1*,2 inactivating mutations.

PARP Inhibitor	Cancer Type	Co-Target	Co-Treatment	Phase	Register Number
Olaparib	BC, OC, FTC, EndA, UCC	mTORC1/2 or AKT	Vistusertib or Capivasertib	1b	NCT02208375
Olaparib	OC, FTC, PPC	CTLA-4	Tremelimumab	2	NCT02571725
Talazoparib	TNBC	mTOR/PI3K	Gedatolisib	2	NCT03911973
Olaparib	BC	CDK4,6 and HR	Palbociclib, Fulvestrant	1	NCT03685331
Niraparib	FTC, OC, EndA, PPC	PI3K	Copanlisib	1	NCT03586661
Olaparib	TNBC	PD-L1	Durvalumab	2	NCT05209529
Niraparib	PanC	PD-1	Dostarlimab	2	NCT04493060
Talazoparib	melanoma	PD-1	Nivolumab	2	NCT04187833
Niraparib	rare tumors	PD-1	Sintilimab	2	NCT04423185
Olaparib	BC	VEGFR or ATR	Cediranib or Ceralasertib	2	NCT04090567
Fluzoparib	HER2^-^ BC	VEGFR	Apatinib	3	NCT04296370
Olaparib	OC, FTC, genital neoplasms	multiple receptor tyrosine kinases	Anlotinib	2	NCT04566952
Olaparib	serous OC	ATR	Ceralasertib	2	NCT03462342
Rucaparib	mesothelioma	—	—	2	NCT03654833
Olaparib	Pt-resistant OC	CDK4,6	Abemaciclib	1/1b	NCT04633239
Olaparib	prostate cancer	LRHL	Leuprolide	2	NCT05498272
Talazoparib	OC and other	BRD2,3,4	ZEN-3694	2	NCT05327010
Veliparib	BC	*—*	Temozolomide	2	NCT01009788

BC—breast cancer, EndA—endometrial adenocarcinoma, FTC—fallopian tube cancer, OC—ovarian cancer, PanC—pancreatic cancer, PPC—primary peritoneal cancer, Pt—platinum-based chemotherapy, TNBC—triple negative breast cancer, UCC—uterine corpus carcinoma.

## Data Availability

Not applicable.

## Supplementary Materials

The following supporting information can be downloaded at https://www.mdpi.com/article/10.3390/ijms24054982/s1.

## References

[1] Hall J.M., Lee M.K., Newman B., Morrow J.E., Anderson L.A., Huey B., King M.C. (1990). Linkage of Early-Onset Familial Breast Cancer to Chromosome 17q21. Science.

[2] Wooster R., Neuhausen S.L., Mangion J., Quirk Y., Ford D., Collins N., Nguyen K., Seal S., Tran T., Averill D. (1994). Localization of a Breast Cancer Susceptibility Gene, BRCA2, to Chromosome 13q12-13. Science.

[3] King T.A., Li W., Brogi E., Yee C.J., Gemignani M.L., Olvera N., Levine D.A., Norton L., Robson M.E., Offit K. (2007). Heterogenic Loss of the Wild-Type BRCA Allele in Human Breast Tumorigenesis. Ann. Surg. Oncol..

[4] Martins F.C., De S., Almendro V., Gönen M., Park S.Y., Blum J.L., Herlihy W., Ethington G., Schnitt S.J., Tung N. (2012). Evolutionary Pathways in BRCA1-Associated Breast Tumors. Cancer Discov..

[5] Kotoula V., Fostira F., Papadopoulou K., Apostolou P., Tsolaki E., Lazaridis G., Manoussou K., Zagouri F., Pectasides D., Vlachos I. (2017). The Fate of BRCA1-Related Germline Mutations in Triple-Negative Breast Tumors. Am. J. Cancer Res..

[6] De Talhouet S., Peron J., Vuilleumier A., Friedlaender A., Viassolo V., Ayme A., Bodmer A., Treilleux I., Lang N., Tille J.-C. (2020). Clinical Outcome of Breast Cancer in Carriers of BRCA1 and BRCA2 Mutations according to Molecular Subtypes. Sci. Rep..

[7] Incorvaia L., Fanale D., Bono M., Calò V., Fiorino A., Brando C., Corsini L.R., Cutaia S., Cancelliere D., Pivetti A. (2020). BRCA1/2 Pathogenic Variants in Triple-Negative versus Luminal-like Breast Cancers: Genotype-Phenotype Correlation in a Cohort of 531 Patients. Ther. Adv. Med. Oncol..

[8] Petrucelli N., Daly M.B., Pal T. (2022). BRCA1- and BRCA2-Associated Hereditary Breast and Ovarian Cancer.

[9] Marchetti C., Ataseven B., Cassani C., Sassu C.M., Congedo L., D’Indinosante M., Cappuccio S., Rhiem K., Hahnen E., Lucci Cordisco E. (2023). Ovarian Cancer Onset across Different BRCA Mutation Types: A View to a More Tailored Approach for BRCA Mutated Patients. Int. J. Gynecol. Cancer.

[10] Yang Q., Yoshimura G., Nakamura M., Nakamura Y., Suzuma T., Umemura T., Mori I., Sakurai T., Kakudo K. (2002). BRCA1 in Non-Inherited Breast Carcinomas (Review). Oncol. Rep..

[11] Wilson C.A., Ramos L., Villaseñor M.R., Anders K.H., Press M.F., Clarke K., Karlan B., Chen J.J., Scully R., Livingston D. (1999). Localization of Human BRCA1 and Its Loss in High-Grade, Non-Inherited Breast Carcinomas. Nat. Genet..

[12] Neff R.T., Senter L., Salani R. (2017). BRCA Mutation in Ovarian Cancer: Testing, Implications and Treatment Considerations. Ther. Adv. Med. Oncol..

[13] Lee Y.-C., Lee Y.-L., Li C.-Y. (2021). BRCA Genes and Related Cancers: A Meta-Analysis from Epidemiological Cohort Studies. Medicina.

[14] Lee Y.-C., Lee Y.-C., Li C.-Y., Lee Y.-L., Chen B.-L. (2020). BRCA1 and BRCA2 Gene Mutations and Lung Cancer Sisk: A Meta-Analysis. Medicina.

[15] Baretta Z., Mocellin S., Goldin E., Olopade O.I., Huo D. (2016). Effect of BRCA Germline Mutations on Breast Cancer Prognosis: A Systematic Review and Meta-Analysis. Medicina.

[16] Gilks C.B., Prat J. (2009). Ovarian Carcinoma Pathology and Genetics: Recent Advances. Hum. Pathol..

[17] McCluggage W.G. (2011). Morphological Subtypes of Ovarian Carcinoma: A Review with Emphasis on New Developments and Pathogenesis. Pathology.

[18] Kim S.I., Lee M., Kim H.S., Chung H.H., Kim J.-W., Park N.H., Song Y.-S. (2019). Effect of BRCA Mutational Status on Survival Outcome in Advanced-Stage High-Grade Serous Ovarian Cancer. J. Ovarian Res..

[19] Risch H.A., McLaughlin J.R., Cole D.E., Rosen B., Bradley L., Kwan E., Jack E., Vesprini D.J., Kuperstein G., Abrahamson J.L. (2001). Prevalence and Penetrance of Germline BRCA1 and BRCA2 Mutations in a Population Series of 649 Women with Ovarian Cancer. Am. J. Hum. Genet..

[20] Zhang S., Royer R., Li S., McLaughlin J.R., Rosen B., Risch H.A., Fan I., Bradley L., Shaw P.A., Narod S.A. (2011). Frequencies of BRCA1 and BRCA2 Mutations among 1,342 Unselected Patients with Invasive Ovarian Cancer. Gynecol. Oncol..

[21] Ramus S.J., Gayther S.A. (2009). The Contribution of BRCA1 and BRCA2 to Ovarian Cancer. Mol. Oncol..

[22] Kuchenbaecker K.B., Hopper J.L., Barnes D.R., Phillips K.-A., Mooij T.M., Roos-Blom M.-J., Jervis S., van Leeuwen F.E., Milne R.L., Andrieu N. (2017). Risks of Breast, Ovarian, and Contralateral Breast Cancer for BRCA1 and BRCA2 Mutation Carriers. JAMA.

[23] Soegaard M., Kjaer S.K., Cox M., Wozniak E., Høgdall E., Høgdall C., Blaakaer J., Jacobs I.J., Gayther S.A., Ramus S.J. (2008). BRCA1 and BRCA2 Mutation Prevalence and Clinical Characteristics of a Population-Based Series of Ovarian Cancer Cases from Denmark. Clin. Cancer Res..

[24] Alsop K., Fereday S., Meldrum C., deFazio A., Emmanuel C., George J., Dobrovic A., Birrer M.J., Webb P.M., Stewart C. (2012). BRCA Mutation Frequency and Patterns of Treatment Response in BRCA Mutation-Positive Women with Ovarian Cancer: A Report from the Australian Ovarian Cancer Study Group. J. Clin. Oncol..

[25] Norquist B.M., Brady M.F., Harrell M.I., Walsh T., Lee M.K., Gulsuner S., Bernards S.S., Casadei S., Burger R.A., Tewari K.S. (2018). Mutations in Homologous Recombination Genes and Outcomes in Ovarian Carcinoma Patients in GOG 218: An NRG Oncology/Gynecologic Oncology Group Study. Clin. Cancer Res..

[26] Tan D.S.P., Rothermundt C., Thomas K., Bancroft E., Eeles R., Shanley S., Ardern-Jones A., Norman A., Kaye S.B., Gore M.E. (2008). “BRCAness” Syndrome in Ovarian Cancer: A Case-Control Study Describing the Clinical Features and Outcome of Patients with Epithelial Ovarian Cancer Associated with BRCA1 and BRCA2 Mutations. J. Clin. Oncol..

[27] Eoh K.J., Kim H.M., Lee J.-Y., Kim S., Kim S.W., Kim Y.T., Nam E.J. (2020). Mutation Landscape of Germline and Somatic BRCA1/2 in Patients with High-Grade Serous Ovarian Cancer. BMC Cancer.

[28] Hennessy B.T.J., Timms K.M., Carey M.S., Gutin A., Meyer L.A., Flake D.D., Abkevich V., Potter J., Pruss D., Glenn P. (2010). Somatic Mutations in BRCA1 and BRCA2 Could Expand the Number of Patients That Benefit from Poly (ADP Ribose) Polymerase Inhibitors in Ovarian Cancer. J. Clin. Oncol..

[29] Ledermann J., Harter P., Gourley C., Friedlander M., Vergote I., Rustin G., Scott C., Meier W., Shapira-Frommer R., Safra T. (2012). Olaparib Maintenance Therapy in Platinum-Sensitive Relapsed Ovarian Cancer. N. Engl. J. Med..

[30] Pennington K.P., Walsh T., Harrell M.I., Lee M.K., Pennil C.C., Rendi M.H., Thornton A., Norquist B.M., Casadei S., Nord A.S. (2014). Germline and Somatic Mutations in Homologous Recombination Genes Predict Platinum Response and Survival in Ovarian, Fallopian Tube, and Peritoneal Carcinomas. Clin. Cancer Res..

[31] Moschetta M., George A., Kaye S.B., Banerjee S. (2016). BRCA Somatic Mutations and Epigenetic BRCA Modifications in Serous Ovarian Cancer. Ann. Oncol..

[32] (2011). Cancer Genome Atlas Research Network; Integrated Genomic Analyses of Ovarian Carcinoma. Nature.

[33] Ledermann J., Harter P., Gourley C., Friedlander M., Vergote I., Rustin G., Scott C.L., Meier W., Shapira-Frommer R., Safra T. (2014). Olaparib Maintenance Therapy in Patients with Platinum-Sensitive Relapsed Serous Ovarian Cancer: A Preplanned Retrospective Analysis of Outcomes by BRCA Status in a Randomised Phase 2 Trial. Lancet Oncol..

[34] Mehrgou A., Akouchekian M. (2016). The Importance of BRCA1 and BRCA2 Genes Mutations in Breast Cancer Development. Med. J. Islam. Repub. Iran.

[35] Aysola K., Desai A., Welch C., Xu J., Qin Y., Reddy V., Matthews R., Owens C., Okoli J., Beech D.J. (2013). Triple Negative Breast Cancer—An Overview. Hered. Genet..

[36] Kotsopoulos J. (2018). BRCA Mutations and Breast Cancer Prevention. Cancers.

[37] Meric-Bernstam F., Brusco L., Daniels M., Wathoo C., Bailey A.M., Strong L., Shaw K., Lu K., Qi Y., Zhao H. (2016). Incidental Germline Variants in 1000 Advanced Cancers on a Prospective Somatic Genomic Profiling Protocol. Ann. Oncol..

[38] Tutt A., Tovey H., Cheang M.C.U., Kernaghan S., Kilburn L., Gazinska P., Owen J., Abraham J., Barrett S., Barrett-Lee P. (2018). Carboplatin in BRCA1/2-Mutated and Triple-Negative Breast Cancer BRCAness Subgroups: The TNT Trial. Nat. Med..

[39] Winter C., Nilsson M.P., Olsson E., George A.M., Chen Y., Kvist A., Törngren T., Vallon-Christersson J., Hegardt C., Häkkinen J. (2016). Targeted Sequencing of BRCA1 and BRCA2 across a Large Unselected Breast Cancer Cohort Suggests That One-Third of Mutations Are Somatic. Ann. Oncol..

[40] den Brok W.D., Schrader K.A., Sun S., Tinker A.V., Zhao E.Y., Aparicio S., Gelmon K.A. (2017). Homologous Recombination Deficiency in Breast Cancer: A Clinical Review. JCO Precis. Oncol..

[41] Bodily W.R., Shirts B.H., Walsh T., Gulsuner S., King M.-C., Parker A., Roosan M., Piccolo S.R. (2020). Effects of Germline and Somatic Events in Candidate BRCA-like Genes on Breast-Tumor Signatures. PLoS ONE.

[42] Turner N., Tutt A., Ashworth A. (2004). Hallmarks of “BRCAness” in Sporadic Cancers. Nat. Rev. Cancer.

[43] Rechsteiner M., Dedes K., Fink D., Pestalozzi B., Sobottka B., Moch H., Wild P., Varga Z. (2018). Somatic BRCA1 Mutations in Clinically Sporadic Breast Cancer with Medullary Histological Features. J. Cancer Res. Clin. Oncol..

[44] Greer J.B., Whitcomb D.C. (2007). Role of BRCA1 and BRCA2 Mutations in Pancreatic Cancer. Gut.

[45] Kowalewski A., Szylberg Ł., Saganek M., Napiontek W., Antosik P., Grzanka D. (2018). Emerging Strategies in BRCA-Positive Pancreatic Cancer. J. Cancer Res. Clin. Oncol..

[46] Luo G., Lu Y., Jin K., Cheng H., Guo M., Liu Z., Long J., Liu C., Ni Q., Yu X. (2015). Pancreatic Cancer: BRCA Mutation and Personalized Treatment. Expert Rev. Anticancer Ther..

[47] Castro E., Eeles R. (2012). The Role of BRCA1 and BRCA2 in Prostate Cancer. Asian J. Androl..

[48] Messina C., Cattrini C., Soldato D., Vallome G., Caffo O., Castro E., Olmos D., Boccardo F., Zanardi E. (2020). BRCA Mutations in Prostate Cancer: Prognostic and Predictive Implications. J. Oncol..

[49] Narod S.A., Neuhausen S., Vichodez G., Armel S., Lynch H.T., Ghadirian P., Cummings S., Olopade O., Stoppa-Lyonnet D., Couch F. (2008). Rapid Progression of Prostate Cancer in Men with a BRCA2 Mutation. Br. J. Cancer.

[50] Pal T., Vadaparampil S., Betts J., Miree C., Li S., Narod S.A. (2008). BRCA1/2 in High-Risk African American Women with Breast Cancer: Providing Genetic Testing through Various Recruitment Strategies. Genet. Test..

[51] Ferla R., Calò V., Cascio S., Rinaldi G., Badalamenti G., Carreca I., Surmacz E., Colucci G., Bazan V., Russo A. (2007). Founder Mutations in BRCA1 and BRCA2 Genes. Ann. Oncol..

[52] Roa B.B., Boyd A.A., Volcik K., Richards C.S. (1996). Ashkenazi Jewish Population Frequencies for Common Mutations in BRCA1 and BRCA2. Nat. Genet..

[53] Janavičius R. (2010). Founder BRCA1/2 Mutations in the Europe: Implications for Hereditary Breast-Ovarian Cancer Prevention and Control. EPMA J..

[54] Sokolenko A.P., Sokolova T.N., Ni V.I., Preobrazhenskaya E.V., Iyevleva A.G., Aleksakhina S.N., Romanko A.A., Bessonov A.A., Gorodnova T.V., Anisimova E.I. (2020). Frequency and Spectrum of Founder and Non-Founder BRCA1 and BRCA2 Mutations in a Large Series of Russian Breast Cancer and Ovarian Cancer Patients. Breast Cancer Res. Treat..

[55] Suspitsin E.N., Sherina N.Y., Ponomariova D.N., Sokolenko A.P., Iyevleva A.G., Gorodnova T.V., Zaitseva O.A., Yatsuk O.S., Togo A.V., Tkachenko N.N. (2009). High Frequency of BRCA1, but Not CHEK2 or NBS1 (NBN), Founder Mutations in Russian Ovarian Cancer Patients. Hered. Cancer Clin. Pract..

[56] Day M., Rappas M., Ptasinska K., Boos D., Oliver A.W., Pearl L.H. (2018). BRCT Domains of the DNA Damage Checkpoint Proteins TOPBP1/Rad4 Display Distinct Specificities for Phosphopeptide Ligands. Elife.

[57] Koonin E.V., Altschul S.F., Bork P. (1996). BRCA1 Protein Products... Functional Motifs. Nat. Genet..

[58] Fabian D., Flatt T. (2011). The Evolution of Aging.

[59] Ben-Aharon I., Levi M., Margel D., Yerushalmi R., Rizel S., Perry S., Sharon E., Hasky N., Abir R., Fisch B. (2018). Premature Ovarian Aging in BRCA Carriers: A Prototype of Systemic Precocious Aging?. Oncotarget.

[60] Kępczyński Ł., Połatyńska K., Nykel A., Sałamunia J., Kałużewski T., Kużawczyk A., Gach A. (2020). Age of Natural Menopause Onset in BRCA1/2 Carriers—Systematic Review and Meta-Analysis. Prz. Menopauzalny.

[61] Drechsel K.C.E., van Tilborg T.C., Eijkemans M.J.C., Lentjes E.G.W.M., Homminga I., Goddijn M., van Golde R.J.T., Verpoest W., Lichtenbelt K., Broekmans J.M. (2022). The Impact of BRCA1- and BRCA2 Mutations on Ovarian Reserve Status. Reprod. Sci..

[62] Semmler L., Reiter-Brennan C., Klein A. (2019). BRCA1 and Breast Cancer: A Review of the Underlying Mechanisms Resulting in the Tissue-Specific Tumorigenesis in Mutation Carriers. J. Breast Cancer.

[63] Roy R., Chun J., Powell S.N. (2011). BRCA1 and BRCA2: Different Roles in a Common Pathway of Genome Protection. Nat. Rev. Cancer.

[64] Schaefer M.H., Serrano L. (2016). Cell Type-Specific Properties and Environment Shape Tissue Specificity of Cancer Genes. Sci. Rep..

[65] Ade C., Roy-Engel A.M., Deininger P.L. (2013). Alu Elements: An Intrinsic Source of Human Genome Instability. Curr. Opin. Virol..

[66] Welcsh P.L., King M.C. (2001). BRCA1 and BRCA2 and the Genetics of Breast and Ovarian Cancer. Hum. Mol. Genet..

[67] Smith T.M., Lee M.K., Szabo C.I., Jerome N., McEuen M., Taylor M., Hood L., King M.C. (1996). Complete Genomic Sequence and Analysis of 117 Kb of Human DNA Containing the Gene BRCA1. Genome Res..

[68] Montagna M., Santacatterina M., Torri A., Menin C., Zullato D., Chieco-Bianchi L., D’Andrea E. (1999). Identification of a 3 Kb Alu-Mediated BRCA1 Gene Rearrangement in Two Breast/Ovarian Cancer Families. Oncogene.

[69] Girolimetti G., Perrone A.M., Santini D., Barbieri E., Guerra F., Ferrari S., Zamagni C., De Iaco P., Gasparre G., Turchetti D. (2014). BRCA-Associated Ovarian Cancer: From Molecular Genetics to Risk Management. Biomed. Res. Int..

[70] Sen S.K., Han K., Wang J., Lee J., Wang H., Callinan P.A., Dyer M., Cordaux R., Liang P., Batzer M.A. (2006). Human Genomic Deletions Mediated by Recombination between Alu Elements. Am. J. Hum. Genet..

[71] Bozsik A., Pócza T., Papp J., Vaszkó T., Butz H., Patócs A., Oláh E. (2020). Complex Characterization of Germline Large Genomic Rearrangements of the BRCA1 and BRCA2 Genes in High-Risk Breast Cancer Patients-Novel Variants from a Large National Center. Int. J. Mol. Sci..

[72] Unger M.A., Nathanson K.L., Calzone K., Antin-Ozerkis D., Shih H.A., Martin A.M., Lenoir G.M., Mazoyer S., Weber B.L. (2000). Screening for Genomic Rearrangements in Families with Breast and Ovarian Cancer Identifies BRCA1 Mutations Previously Missed by Conformation-Sensitive Gel Electrophoresis or Sequencing. Am. J. Hum. Genet..

[73] Nordling M., Karlsson P., Wahlström J., Engwall Y., Wallgren A., Martinsson T. (1998). A Large Deletion Disrupts the Exon 3 Transcription Activation Domain of the BRCA2 Gene in a Breast/Ovarian Cancer Family. Cancer Res..

[74] Ewald I.P., Ribeiro P.L.I., Palmero E.I., Cossio S.L., Giugliani R., Ashton-Prolla P. (2009). Genomic Rearrangements in BRCA1 and BRCA2: A Literature Review. Genet. Mol. Biol..

[75] Li W.-F., Hu Z., Rao N.-Y., Song C.-G., Zhang B., Cao M.-Z., Su F.-X., Wang Y.-S., He P.-Q., Di G.-H. (2008). The Prevalence of BRCA1 and BRCA2 Germline Mutations in High-Risk Breast Cancer Patients of Chinese Han Nationality: Two Recurrent Mutations Were Identified. Breast Cancer Res. Treat..

[76] Fachal L., Blanco A., Santamariña M., Carracedo A., Vega A. (2014). Large Genomic Rearrangements of BRCA1 and BRCA2 among Patients Referred for Genetic Analysis in Galicia (NW Spain): Delimitation and Mechanism of Three Novel BRCA1 Rearrangements. PLoS ONE.

[77] Lou D.I., McBee R.M., Le U.Q., Stone A.C., Wilkerson G.K., Demogines A.M., Sawyer S.L. (2014). Rapid Evolution of BRCA1 and BRCA2 in Humans and Other Primates. BMC Evol. Biol..

[78] O’Donovan P.J., Livingston D.M. (2010). BRCA1 and BRCA2: Breast/Ovarian Cancer Susceptibility Gene Products and Participants in DNA Double-Strand Break Repair. Carcinogenesis.

[79] Hemel D., Domchek S.M. (2010). Breast Cancer Predisposition Syndromes. Hematol. Oncol. Clin. N. Am..

[80] Preisler-Adams S., Schönbuchner I., Fiebig B., Welling B., Dworniczak B., Weber B.H.F. (2006). Gross Rearrangements in BRCA1 but Not BRCA2 Play a Notable Role in Predisposition to Breast and Ovarian Cancer in High-Risk Families of German Origin. Cancer Genet. Cytogenet..

[81] Rohlfs E.M., Puget N., Graham M.L., Weber B.L., Garber J.E., Skrzynia C., Halperin J.L., Lenoir G.M., Silverman L.M., Mazoyer S. (2000). An Alu-Mediated 7.1 Kb Deletion of BRCA1 Exons 8 and 9 in Breast and Ovarian Cancer Families That Results in Alternative Splicing of Exon 10. Genes Chromosom. Cancer.

[82] Agata S., Dalla Palma M., Callegaro M., Scaini M.C., Menin C., Ghiotto C., Nicoletto O., Zavagno G., Chieco-Bianchi L., D’Andrea E. (2005). Large Genomic Deletions Inactivate the BRCA2 Gene in Breast Cancer Families. J. Med. Genet..

[83] Karhu R., Laurila E., Kallioniemi A., Syrjäkoski K. (2006). Large Genomic BRCA2 Rearrangements and Male Breast Cancer. Cancer Detect. Prev..

[84] Woodward A.M., Davis T.A., Silva A.G.S., Kirk J.A., Leary J.A. (2005). kConFab Investigators Large Genomic Rearrangements of Both BRCA2 and BRCA1 Are a Feature of the Inherited Breast/Ovarian Cancer Phenotype in Selected Families. J. Med. Genet..

[85] Peixoto A., Santos C., Rocha P., Pinto P., Bizarro S., Teixeira M.R. (2009). Molecular Diagnosis of the Portuguese Founder Mutation BRCA2 c.156_157insAlu. Breast Cancer Res. Treat..

[86] Duncan J.A., Reeves J.R., Cooke T.G. (1998). BRCA1 and BRCA2 Proteins: Roles in Health and Disease. Mol. Pathol..

[87] Brzovic P.S., Rajagopal P., Hoyt D.W., King M.-C., Klevit R.E. (2001). Structure of a BRCA1–BARD1 Heterodimeric RING–RING Complex. Nat. Struct. Mol. Biol..

[88] Wang Y., Bernhardy A.J., Johnson N. (2017). Abstract A23: BRCA1 Mutations in the BRCT Domain Can Be Removed through Alternative Splicing and Induce PARP Inhibitor Resistance. Mol. Cancer Res..

[89] Wu W., Koike A., Takeshita T., Ohta T. (2008). The Ubiquitin E3 Ligase Activity of BRCA1 and Its Biological Functions. Cell Div..

[90] Hashizume R., Fukuda M., Maeda I., Nishikawa H., Oyake D., Yabuki Y., Ogata H., Ohta T. (2001). The RING Heterodimer BRCA1-BARD1 Is a Ubiquitin Ligase Inactivated by a Breast Cancer-Derived Mutation. J. Biol. Chem..

[91] Birrane G., Varma A.K., Soni A., Ladias J.A.A. (2007). Crystal Structure of the BARD1 BRCT Domains. Biochemistry.

[92] Jin Y., Xu X.L., Yang M.C., Wei F., Ayi T.C., Bowcock A.M., Baer R. (1997). Cell Cycle-Dependent Colocalization of BARD1 and BRCA1 Proteins in Discrete Nuclear Domains. Proc. Natl. Acad. Sci. USA.

[93] Wang B., Matsuoka S., Ballif B.A., Zhang D., Smogorzewska A., Gygi S.P., Elledge S.J. (2007). Abraxas and RAP80 Form a BRCA1 Protein Complex Required for the DNA Damage Response. Science.

[94] Mittenberg A.G., Moiseeva T.N., Barlev N.A. (2008). Role of Proteasomes in Transcription and Their Regulation by Covalent Modifications. Front. Biosci..

[95] Paull T.T., Cortez D., Bowers B., Elledge S.J., Gellert M. (2001). Direct DNA Binding by Brca1. Proc. Natl. Acad. Sci. USA.

[96] Starita L.M., Parvin J.D. (2003). The Multiple Nuclear Functions of BRCA1: Transcription, Ubiquitination and DNA Repair. Curr. Opin. Cell Biol..

[97] Zheng L., Pan H., Li S., Flesken-Nikitin A., Chen P.L., Boyer T.G., Lee W.H. (2000). Sequence-Specific Transcriptional Corepressor Function for BRCA1 through a Novel Zinc Finger Protein, ZBRK1. Mol. Cell.

[98] Clark S.L., Rodriguez A.M., Snyder R.R., Hankins G.D.V., Boehning D. (2012). Structure-Function of the Tumor Suppressor BRCA1. Comput. Struct. Biotechnol. J..

[99] Lee M.S., Green R., Marsillac S.M., Coquelle N., Williams R.S., Yeung T., Foo D., Hau D.D., Hui B., Monteiro A.N.A. (2010). Comprehensive Analysis of Missense Variations in the BRCT Domain of BRCA1 by Structural and Functional Assays. Cancer Res..

[100] Williams R.S., Bernstein N., Lee M.S., Rakovszky M.L., Cui D., Green R., Weinfeld M., Glover J.N.M. (2005). Structural Basis for Phosphorylation-Dependent Signaling in the DNA-Damage Response. Biochem. Cell Biol..

[101] Zhang J., Willers H., Feng Z., Ghosh J.C., Kim S., Weaver D.T., Chung J.H., Powell S.N., Xia F. (2004). Chk2 Phosphorylation of BRCA1 Regulates DNA Double-Strand Break Repair. Mol. Cell. Biol..

[102] Matsuoka S., Ballif B.A., Smogorzewska A., McDonald E.R., Hurov K.E., Luo J., Bakalarski C.E., Zhao Z., Solimini N., Lerenthal Y. (2007). ATM and ATR Substrate Analysis Reveals Extensive Protein Networks Responsive to DNA Damage. Science.

[103] Reinhardt H.C., Yaffe M.B. (2009). Kinases That Control the Cell Cycle in Response to DNA Damage: Chk1, Chk2, and MK2. Curr. Opin. Cell Biol..

[104] Witus S.R., Burrell A.L., Farrell D.P., Kang J., Wang M., Hansen J.M., Pravat A., Tuttle L.M., Stewart M.D., Brzovic P.S. (2021). BRCA1/BARD1 Site-Specific Ubiquitylation of Nucleosomal H2A Is Directed by BARD1. Nat. Struct. Mol. Biol..

[105] Hu Q., Botuyan M.V., Zhao D., Cui G., Mer E., Mer G. (2021). Mechanisms of BRCA1-BARD1 Nucleosome Recognition and Ubiquitylation. Nature.

[106] Becker J.R., Clifford G., Bonnet C., Groth A., Wilson M.D., Chapman J.R. (2021). BARD1 Reads H2A Lysine 15 Ubiquitination to Direct Homologous Recombination. Nature.

[107] Yang H., Jeffrey P.D., Miller J., Kinnucan E., Sun Y., Thoma N.H., Zheng N., Chen P.-L., Lee W.-H., Pavletich N.P. (2002). BRCA2 Function in DNA Binding and Recombination from a BRCA2-DSS1-SsDNA Structure. Science.

[108] Trego K.S., Groesser T., Davalos A.R., Parplys A.C., Zhao W., Nelson M.R., Hlaing A., Shih B., Rydberg B., Pluth J.M. (2016). Non-Catalytic Roles for XPG with BRCA1 and BRCA2 in Homologous Recombination and Genome Stability. Mol. Cell.

[109] Scully R., Livingston D.M. (2000). In Search of the Tumour-Suppressor Functions of BRCA1 and BRCA2. Nature.

[110] Deng C.X., Scott F. (2000). Role of the Tumor Suppressor Gene Brca1 in Genetic Stability and Mammary Gland Tumor Formation. Oncogene.

[111] Foray N., Marot D., Randrianarison V., Venezia N.D., Picard D., Perricaudet M., Favaudon V., Jeggo P. (2002). Constitutive Association of BRCA1 and C-Abl and Its ATM-Dependent Disruption after Irradiation. Mol. Cell. Biol..

[112] Levav-Cohen Y., Goldberg Z., Zuckerman V., Grossman T., Haupt S., Haupt Y. (2005). C-Abl as a Modulator of P53. Biochem. Biophys. Res. Commun..

[113] Hantschel O., Rix U., Schmidt U., Bürckstümmer T., Kneidinger M., Schütze G., Colinge J., Bennett K.L., Ellmeier W., Valent P. (2007). The Btk Tyrosine Kinase Is a Major Target of the Bcr-Abl Inhibitor Dasatinib. Proc. Natl. Acad. Sci. USA.

[114] Althubiti M., Rada M., Samuel J., Escorsa J.M., Najeeb H., Lee K.-G., Lam K.-P., Jones G.D.D., Barlev N.A., Macip S. (2016). BTK Modulates P53 Activity to Enhance Apoptotic and Senescent Responses. Cancer Res..

[115] Rada M., Barlev N., Macip S. (2018). BTK: A Two-Faced Effector in Cancer and Tumour Suppression. Cell Death Dis..

[116] Xu X., Qiao W., Linke S.P., Cao L., Li W.M., Furth P.A., Harris C.C., Deng C.X. (2001). Genetic Interactions between Tumor Suppressors Brca1 and P53 in Apoptosis, Cell Cycle and Tumorigenesis. Nat. Genet..

[117] Zhang H., Somasundaram K., Peng Y., Tian H., Zhang H., Bi D., Weber B.L., El-Deiry W.S. (1998). BRCA1 Physically Associates with P53 and Stimulates Its Transcriptional Activity. Oncogene.

[118] Pietrasik S., Zajac G., Morawiec J., Soszynski M., Fila M., Blasiak J. (2020). Interplay between BRCA1 and GADD45A and Its Potential for Nucleotide Excision Repair in Breast Cancer Pathogenesis. Int. J. Mol. Sci..

[119] Evers B., Jonkers J. (2006). Mouse Models of BRCA1 and BRCA2 Deficiency: Past Lessons, Current Understanding and Future Prospects. Oncogene.

[120] Clarke C.L., Sandle J., Jones A.A., Sofronis A., Patani N.R., Lakhani S.R. (2006). Mapping Loss of Heterozygosity in Normal Human Breast Cells from BRCA1/2 Carriers. Br. J. Cancer.

[121] Armes J.E., Egan A.J.M., Southey M.C., Dite G.S., McCredie M.R.E., Giles G.G., Hopper J.L., Venter D.J. (1998). The Histologic Phenotypes of Breast Carcinoma Occurring before Age 40 Years in Women with and without BRCA1 or BRCA2 Germline Mutations. Cancer.

[122] Mote P.A., Leary J.A., Avery K.A., Sandelin K., Chenevix-Trench G., Kirk J.A., Clarke C.L. (2004). Germ-Line Mutations in BRCA1 or BRCA2 in the Normal Breast Are Associated with Altered Expression of Estrogen-Responsive Proteins and the Predominance of Progesterone Receptor A. Genes Chromosom. Cancer.

[123] Ingthorsson S., Traustadottir G.A., Gudjonsson T. (2022). Cellular Plasticity and Heterotypic Interactions during Breast Morphogenesis and Cancer Initiation. Cancers.

[124] Avşar Abdik E. (2021). Differentiated Pre-Adipocytes Promote Proliferation, Migration and Epithelial-Mesenchymal Transition in Breast Cancer Cells of Different P53 Status. Mol. Biol. Rep..

[125] Choi J., Cha Y.J., Koo J.S. (2018). Adipocyte Biology in Breast Cancer: From Silent Bystander to Active Facilitator. Prog. Lipid Res..

[126] Takehara M., Sato Y., Kimura T., Noda K., Miyamoto H., Fujino Y., Miyoshi J., Nakamura F., Wada H., Bando Y. (2020). Cancer-Associated Adipocytes Promote Pancreatic Cancer Progression through SAA1 Expression. Cancer Sci..

[127] Kothari C., Diorio C., Durocher F. (2020). The Importance of Breast Adipose Tissue in Breast Cancer. Int. J. Mol. Sci..

[128] Yao H., He S. (2021). Multi-faceted Role of Cancer-associated Adipocytes in the Tumor Microenvironment (Review). Mol. Med. Rep..

[129] Jafari N., Kolla M., Meshulam T., Shafran J.S., Qiu Y., Casey A.N., Pompa I.R., Ennis C.S., Mazzeo C.S., Rabhi N. (2021). Adipocyte-Derived Exosomes May Promote Breast Cancer Progression in Type 2 Diabetes. Sci. Signal..

[130] Lee Y., Jung W.H., Koo J.S. (2015). Adipocytes Can Induce Epithelial-Mesenchymal Transition in Breast Cancer Cells. Breast Cancer Res. Treat..

[131] Xie Y., Wang B., Zhao Y., Tao Z., Wang Y., Chen G., Hu X. (2022). Mammary Adipocytes Protect Triple-Negative Breast Cancer Cells from Ferroptosis. J. Hematol. Oncol..

[132] Zhou C., He X., Tong C., Li H., Xie C., Wu Y., Wang L., Yan X., Luo D., Tang Y. (2022). Cancer-Associated Adipocytes Promote the Invasion and Metastasis in Breast Cancer through LIF/CXCLs Positive Feedback Loop. Int. J. Biol. Sci..

[133] Jones L.P., Buelto D., Tago E., Owusu-Boaitey K.E. (2011). Abnormal Mammary Adipose Tissue Environment of Brca1 Mutant Mice Show a Persistent Deposition of Highly Vascularized Multilocular Adipocytes. J. Cancer Sci. Ther..

[134] Miran I., Scherer D., Ostyn P., Mazouni C., Drusch F., Bernard M., Louvet E., Adam J., Mathieu M.-C., Haffa M. (2020). Adipose Tissue Properties in Tumor-Bearing Breasts. Front. Oncol..

[135] Koellensperger E., Bonnert L.-C., Zoernig I., Marmé F., Sandmann S., Germann G., Gramley F., Leimer U. (2017). The Impact of Human Adipose Tissue-Derived Stem Cells on Breast Cancer Cells: Implications for Cell-Assisted Lipotransfers in Breast Reconstruction. Stem Cell Res. Ther..

[136] Luo G., He Y., Yu X. (2018). Bone Marrow Adipocyte: An Intimate Partner With Tumor Cells in Bone Metastasis. Front. Endocrinol..

[137] Konstantinopoulos P.A., Ceccaldi R., Shapiro G.I., D’Andrea A.D. (2015). Homologous Recombination Deficiency: Exploiting the Fundamental Vulnerability of Ovarian Cancer. Cancer Discov..

[138] Torrisi R., Zuradelli M., Agostinetto E., Masci G., Losurdo A., De Sanctis R., Santoro A. (2019). Platinum Salts in the Treatment of BRCA-Associated Breast Cancer: A True Targeted Chemotherapy?. Crit. Rev. Oncol. Hematol..

[139] Giannone G., Scotto G., Katsaros D., De Giorgi U., Farolfi A., Borella F., Cosma S., Ferrero A., Mangiacotti S., Villa M. (2021). Hypersensitivity to Platinum Salts According to BRCA Status in Ovarian Cancer: A Retrospective Analysis of Clinical Outcomes and Systematic Review of Literature. Gynecol. Oncol..

[140] Wattenberg M.M., Asch D., Yu S., O’Dwyer P.J., Domchek S.M., Nathanson K.L., Rosen M.A., Beatty G.L., Siegelman E.S., Reiss K.A. (2020). Platinum Response Characteristics of Patients with Pancreatic Ductal Adenocarcinoma and a Germline BRCA1, BRCA2 or PALB2 Mutation. Br. J. Cancer.

[141] Byrski T., Huzarski T., Dent R., Gronwald J., Zuziak D., Cybulski C., Kladny J., Gorski B., Lubinski J., Narod S.A. (2009). Response to Neoadjuvant Therapy with Cisplatin in BRCA1-Positive Breast Cancer Patients. Breast Cancer Res. Treat..

[142] Maxwell K.N., Wubbenhorst B., Wenz B.M., De Sloover D., Pluta J., Emery L., Barrett A., Kraya A.A., Anastopoulos I.N., Yu S. (2017). BRCA Locus-Specific Loss of Heterozygosity in Germline BRCA1 and BRCA2 Carriers. Nat. Commun..

[143] Afghahi A., Timms K.M., Vinayak S., Jensen K.C., Kurian A.W., Carlson R.W., Chang P.-J., Schackmann E., Hartman A.-R., Ford J.M. (2017). Tumor BRCA1 Reversion Mutation Arising during Neoadjuvant Platinum-Based Chemotherapy in Triple-Negative Breast Cancer Is Associated with Therapy Resistance. Clin. Cancer Res..

[144] Pilié P.G., Tang C., Mills G.B., Yap T.A. (2019). State-of-the-Art Strategies for Targeting the DNA Damage Response in Cancer. Nat. Rev. Clin. Oncol..

[145] Mateo J., Lord C.J., Serra V., Tutt A., Balmaña J., Castroviejo-Bermejo M., Cruz C., Oaknin A., Kaye S.B., de Bono J.S. (2019). A Decade of Clinical Development of PARP Inhibitors in Perspective. Ann. Oncol..

[146] Bredow K., Blümcke B., Schneider S., Püsken M., Schmutzler R., Rhiem K. (2022). Long-Term Survival of a BRCA2 Mutation Carrier Following Second Ovarian Cancer Relapse Using PARPi Therapy: A Case Report. Mol. Clin. Oncol..

[147] Wang N., Yu X. (2022). Comparison between Talazoparib and Conventional Chemotherapy in the Treatment of HER2-Positive Breast Cancer Patients: A Retrospective Study. Front. Immunol..

[148] Zong D., Adam S., Wang Y., Sasanuma H., Callén E., Murga M., Day A., Kruhlak M.J., Wong N., Munro M. (2019). BRCA1 Haploinsufficiency Is Masked by RNF168-Mediated Chromatin Ubiquitylation. Mol. Cell.

[149] Sullivan-Reed K., Bolton-Gillespie E., Dasgupta Y., Langer S., Siciliano M., Nieborowska-Skorska M., Hanamshet K., Belyaeva E.A., Bernhardy A.J., Lee J. (2018). Simultaneous Targeting of PARP1 and RAD52 Triggers Dual Synthetic Lethality in BRCA-Deficient Tumor Cells. Cell Rep..

[150] Kayumov M., Jia L., Pardaev A., Song S.-S., Mirzaakhmedov S., Ding C., Cheng Y.-J., Zhang R.I., Bao X., Miao Z.-H. (2022). Design, Synthesis and Pharmacological Evaluation of New PARP1 Inhibitors by Merging Pharmacophores of Olaparib and the Natural Product Alantolactone. Eur. J. Med. Chem..

[151] Sinha S., Chatterjee S., Paul S., Das B., Dash S.R., Das C., Kundu C.N. (2022). Olaparib Enhances the Resveratrol-Mediated Apoptosis in Breast Cancer Cells by Inhibiting the Homologous Recombination Repair Pathway. Exp. Cell Res..

[152] Kim H., Xu H., George E., Hallberg D., Kumar S., Jagannathan V., Medvedev S., Kinose Y., Devins K., Verma P. (2020). Combining PARP with ATR Inhibition Overcomes PARP Inhibitor and Platinum Resistance in Ovarian Cancer Models. Nat. Commun..

[153] Xu H., Hurley L.H. (2022). A First-in-Class Clinical G-Quadruplex-Targeting Drug. The Bench-to-Bedside Translation of the Fluoroquinolone QQ58 to CX-5461 (Pidnarulex). Bioorg. Med. Chem. Lett..

[154] Jackson L.M., Moldovan G.-L. (2022). Mechanisms of PARP1 Inhibitor Resistance and Their Implications for Cancer Treatment. NAR Cancer.

[155] Mustafina O.E. (2013). The Possible Roles of Human Alu Elements in Aging. Front. Genet..

[156] Morales M.E., White T.B., Streva V.A., DeFreece C.B., Hedges D.J., Deininger P.L. (2015). The Contribution of Alu Elements to Mutagenic DNA Double-Strand Break Repair. PLoS Genet..

[157] González-Martín A., Pothuri B., Vergote I., DePont Christensen R., Graybill W., Mirza M.R., McCormick C., Lorusso D., Hoskins P., Freyer G. (2019). Niraparib in Patients with Newly Diagnosed Advanced Ovarian Cancer. N. Engl. J. Med..

[158] Hu Y., Petit S.A., Ficarro S.B., Toomire K.J., Xie A., Lim E., Cao S.A., Park E., Eck M.J., Scully R. (2014). PARP1-Driven Poly-ADP-Ribosylation Regulates BRCA1 Function in Homologous Recombination-Mediated DNA Repair. Cancer Discov..

[159] Lodovichi S., Quadri R., Sertic S., Pellicioli A. (2023). PARylation of BRCA1 Limits DNA Break Resection through BRCA2 and EXO1. Cell Rep..

[160] Tookman L.A., Browne A.K., Connell C.M., Bridge G., Ingemarsdotter C.K., Dowson S., Shibata A., Lockley M., Martin S.A., McNeish I.A. (2016). RAD51 and BRCA2 Enhance Oncolytic Adenovirus Type 5 Activity in Ovarian Cancer. Mol. Cancer Res..

[161] Reisländer T., Lombardi E.P., Groelly F.J., Miar A., Porru M., Di Vito S., Wright B., Lockstone H., Biroccio A., Harris A. (2019). BRCA2 Abrogation Triggers Innate Immune Responses Potentiated by Treatment with PARP Inhibitors. Nat. Commun..

[162] Loizzi V., Dellino M., Cerbone M., Arezzo F., Cazzato G., Damiani G.R., Pinto V., Silvestris E., Kardhashi A., Cicinelli E. (2023). The Role of Hormonal Replacement Therapy in BRCA Mutated Patients: Lights and Shadows. Int. J. Mol. Sci..

[163] Singer C.F. (2021). Nonsurgical Prevention Strategies in BRCA1 and BRCA2 Mutation Carriers. Breast Care.

[164] Nehme R., Diab-Assaf M., Decombat C., Delort L., Caldefie-Chezet F. (2022). Targeting Adiponectin in Breast Cancer. Biomedicines.

[165] Vousden K.H., Prives C. (2009). Blinded by the Light: The Growing Complexity of P53. Cell.

[166] Williamson C.T., Kubota E., Hamill J.D., Klimowicz A., Ye R., Muzik H., Dean M., Tu L., Gilley D., Magliocco A.M. (2012). Enhanced Cytotoxicity of PARP Inhibition in Mantle Cell Lymphoma Harbouring Mutations in Both ATM and P53. EMBO Mol. Med..

[167] Smeby J., Kryeziu K., Berg K.C.G., Eilertsen I.A., Eide P.W., Johannessen B., Guren M.G., Nesbakken A., Bruun J., Lothe R.A. (2020). Molecular Correlates of Sensitivity to PARP Inhibition beyond Homologous Recombination Deficiency in Pre-Clinical Models of Colorectal Cancer Point to Wild-Type TP53 Activity. EBioMedicine.

[168] Hong T., Lei G., Chen X., Li H., Zhang X., Wu N., Zhao Y., Zhang Y., Wang J. (2021). PARP Inhibition Promotes Ferroptosis via Repressing SLC7A11 and Synergizes with Ferroptosis Inducers in BRCA-Proficient Ovarian Cancer. Redox Biol..

[169] Feng Z., Kachnic L., Zhang J., Powell S.N., Xia F. (2004). DNA Damage Induces P53-Dependent BRCA1 Nuclear Export. J. Biol. Chem..

[170] Parfenyev S., Singh A., Fedorova O., Daks A., Kulshreshtha R., Barlev N.A. (2021). Interplay between P53 and Non-Coding RNAs in the Regulation of EMT in Breast Cancer. Cell Death Dis..

[171] Barlev N.A., Sayan B.S., Candi E., Okorokov A.L. (2010). The MicroRNA and P53 Families Join Forces against Cancer. Cell Death Differ..

[172] Lezina L., Aksenova V., Fedorova O., Malikova D., Shuvalov O., Antonov A.V., Tentler D., Garabadgiu A.V., Melino G., Barlev N.A. (2015). KMT Set7/9 Affects Genotoxic Stress Response via the Mdm2 Axis. Oncotarget.

[173] Portman N., Milioli H.H., Alexandrou S., Coulson R., Yong A., Fernandez K.J., Chia K.M., Halilovic E., Segara D., Parker A. (2020). MDM2 Inhibition in Combination with Endocrine Therapy and CDK4/6 Inhibition for the Treatment of ER-Positive Breast Cancer. Breast Cancer Res..

[174] Bulatov E., Sayarova R., Mingaleeva R., Miftakhova R., Gomzikova M., Ignatyev Y., Petukhov A., Davidovich P., Rizvanov A., Barlev N.A. (2018). Isatin-Schiff Base-Copper (II) Complex Induces Cell Death in P53-Positive Tumors. Cell Death Discov..

[175] Davidovich P., Aksenova V., Petrova V., Tentler D., Orlova D., Smirnov S., Gurzhiy V., Okorokov A.L., Garabadzhiu A., Melino G. (2015). Discovery of Novel Isatin-Based P53 Inducers. ACS Med. Chem. Lett..

[176] Fedorova O., Daks A., Petrova V., Petukhov A., Lezina L., Shuvalov O., Davidovich P., Kriger D., Lomert E., Tentler D. (2018). Novel Isatin-Derived Molecules Activate P53 via Interference with Mdm2 to Promote Apoptosis. Cell Cycle.

[177] Gorodnova T.V., Sokolenko A.P., Kotiv K.B., Sokolova T.N., Ivantsov A.O., Guseynov K.D., Nekrasova E.A., Smirnova O.A., Berlev I.V., Imyanitov E.N. (2021). Neoadjuvant Therapy of BRCA1-Driven Ovarian Cancer by Combination of Cisplatin, Mitomycin C and Doxorubicin. Hered. Cancer Clin. Pract..

[178] Fasching P.A., Loibl S., Hu C., Hart S.N., Shimelis H., Moore R., Schem C., Tesch H., Untch M., Hilfrich J. (2018). BRCA1/2 Mutations and Bevacizumab in the Neoadjuvant Treatment of Breast Cancer: Response and Prognosis Results in Patients With Triple-Negative Breast Cancer From the GeparQuinto Study. J. Clin. Oncol..

