# Supplementary material for: BRCA Mutations—The Achilles Heel of Breast, Ovarian and Other Epithelial Cancers
Source: Int J Mol Sci. 2023 Mar 5;24(5):4982. doi: 10.3390/ijms24054982 (PMC10003548; doi:10.3390/ijms24054982)
Supplement: Supplementary file 1 [file ijms-24-04982-s001.zip › ijms-2245973-supplementary.pdf]

Supplementary Table S1. Registered ongoing clinical trials against cancers with inactivating mutations in BRCA1,BRCA2 or other HR-related genes

PARP inhibitor	Cancer type	Mutated genes	Co-treatment (Co-Target)	Phase	Register number
Olaparib	Pt-sensitive relapsed OC	BRCA1,2	NA	3	NCT01874353
-	OC, BC, PC, PanC	-	-	2	NCT01078662
-	OC, FTC, PPC	-	-	1	NCT03943173
-	BC	-	-	3	NCT02000622
-	BC	BRCA1,2	NA (vs Pt)	2	NCT05629429
-	BC	HR (somatic vs germline)	NA	2	NCT03344965
-	post-Pt BC	BRCA1,2	-	3	NCT01844986
-	Pt-sensitive PanC	BRCA1,2	-	2	NCT02184195
-	PanC	BRCA1,2, PALB2	-	2	NCT04858334
-	CRPC	BRCA1,2	-	4	NCT05457257
-	various	HR	-	2	NCT03967938
-	various	-	-	2	NCT03967938
-	biliary tract cancer	-	-	2	NCT0404283
-	Melanoma	BRCA1,2	-	2	NCT05482074
Talazoparib	OC, FTC	BRCA1,2	-	1	NCT04584255
-	BC	-	-	2	NCT03990896
-	BC	-	-	1	NCT03343054
-	BC	HR	-	2	NCT02345265
-	other than OC or BC	HR	-	2	NCT02286687
-	TNBC	BRCA1,2	-	2	NCT04755868
Rucaparib	NSCLC	BRCA1,2 with LOH	-	2	NCT03845296
-	Pt-resistant PanC	BRCA1,2, PALB2	-	2	NCT03140670
-	OC,FTC,PPC	BRCA1,2	-	3	NCT02855944
Niraparib	PC	HR	-	2	NCT04030559
Pamiparib	CRPC	BRCA1,2	-	2	NCT05327621
Pamiparib	Pt-resistant OC	BRCA1,2	-	2	NCT05044871
none	BC, OC, PC, PanC	BRCA1,2, PALB2	Pidnarulex	2	NCT04890613
-	OC, BC	BRCA1,2,	Pembrolizumab	2	NCT03428802
-	PanC	BRCA1,2	Chlorambucil	2	NCT04692740
-	prevention of BC	BRCA1	Denosumab (RANKL)	3	NCT04711109
-	prevention of BC	BRCA1,2	Metformin	3	NCT01905046
-	prevention of BC, OC	BRCA1,2	Letrozole	3	NCT00673335
Olaparib	$HER2^-$ BC	BRCA1,2	Atezolizumab (PD-L1)	2	NCT02849496
Talazoparib	locally advanced or metastatic solid tumors	BRCA1,2, vs ATM	Avelumab (PD-L1)	2	NCT03565991

Talazoparib	UC,OC,TNBC,NSCLC,CRPC	BRCA1,2,ATM	-	1b/2	NCT03330405
Olaparib	serous OC, recurrent FTC	BRCA1,2	Durvalumab (PD-L1) Tremelimumab(CTLA4)	2	NCT02953457
Fluzoparib	HER2$^-$ BC	BRCA1,2	Camrelizumab (PD-1)	2	NCT05576389
Niraparib	BC, PanC, OC, FTC, PPC	BRCA1,2	Dostarlimab (PD-1)	1	NCT04673448
Niraparib	HER2$^-$ BC	BRCA1,2, PALB2	-	1	NCT04584255
Niraparib	PanC	BRCA1,2, PALB2	-	2	NCT04493060
Olaparib	BC, OC, FTC, EndA, UCC	BRCA1,2	Vistusertib (mTORC1/2) or Capivasertib (AKT)	1b	NCT02208375
Olaparib	OC,FTC,PPC	BRCA1,2	Tremelimumab (CTLA-4)	2	NCT02571725
Talazoparib	TNBC	BRCA1,2	Gedatolisib (mTOR/PI3K)	2	NCT03911973
Olaparib	BC	BRCA1,2	Palbociclib (CDK4,6) + Fulvestrant (HR)	1	NCT03685331
Niraparib	FTC,OC,EndA,PPC	BRCA1,2	Copanlisib (PI3K)	1	NCT03586661
Olaparib	TNBC	BRCA1,2	Durvalumab (PD-L1)	2	NCT05209529
Olaparib	HER2$^-$ BC	BRCA1,2	-	2	NCT05498155
Olaparib	BC	BRCA1,2, HR	-	2	NCT05659914
Olaparib	HER2$^-$ ER$^+$ BC	HR	Durvalumab (PD-L1) Fulvestrant (HR)	2	NCT04053322
Rucaparib	BC and OC excluded	HR	Atezolizumab (PD-L1)	2	NCT04276376
Olaparib	PanC	BRCA1,2,PALB2 vs other HR vs non-HR Pt-sensitive	Pembrolizumab (PD-1)	2	NCT04666740
-	Melanoma	HR	-	2	NCT04633902
-	BC	HR	-	2	NCT03025035
-	colorectal cancer	BRCA1,2	-	2	NCT05201612
-	TNBC	BRCA1,2	-	2	NCT05485766

Talazoparib	Melanoma	BRCA1,2	Nivolumab (PD-1)	2	NCT04187833
Niraparib	rare tumors	BRCA1,2,	Sintilimab (PD-1)	2	NCT04423185
Olaparib	BC	BRCA1,2	Cediranib (VEGFR) or Ceralasertib (ATR)	2	NCT04090567
Fluzoparib	HER2- BC	BRCA1	Apatinib (VEGFR)	3	NCT04296370
Olaparib	OC,PPC,FTC	BRCA1,2	Cediranib (VEGFR)		NCT02345265
Olaparib	OC	BRCA1,2	-	2	NCT03117933
Fluzoparib	relapsed OC	BRCA1,2	Apatinib (VEGFR)	2	NCT05479487
Olaparib	OC, FTC, genital neoplasms	BRCA1,2	Anlotinib (multiple RTK)	1	NCT04566952
Olaparib	serous OC	HR	Ceralasertib (ATR)	2	NCT03462342
Rucaparib	Mesothelioma	BRCA1, BAP1	NA	2	NCT03654833
Olaparib	recurrent Pt-resistant OC	BRCA1,2	Abemaciclib (CDK4,6)	1/1b	NCT04633239
Olaparib	PC	BRCA1,2	Leuprolide (LRHL)	2	NCT05498272
Talazoparib	OC and other malignant	BRCA1,2	ZEN-3694 (BET Bromodomai n)	2	NCT05327010
Olaparib	BC	BRCA1,2	Carboplatin Paclitaxel	2/3	NCT03150576
Niraparib	OC, FTC, peritoneal cancer	BRCA1,2	Carboplatin, Paclitaxel, Bevacizumab	3	NCT05009082
Niraparib	OC, FTC, genital neoplasms	BRCA1,2	Bevacizumab (VEGF)	2	NCT04556071
Niraparib	HER2- BC	BRCA1,2, PALB2, CHEK2	Gemcitabine, Cisplatin	2	NCT04508803
Veliparib	BC	BRCA1,2	Temozolomi de	2	NCT01009788
Veliparib	OC	BRCA1,2	Carboplatin, Paclitaxel	3	NCT02470585
Veliparib	HER2- BC	BRCA1,2	Carboplatin Paclitaxel	3	NCT02163694
Veliparib	adult solid tumors in HBOC	BRCA1,2	Carboplatin, Paclitaxel	2	NCT00535119
Veliparib	BC	BRCA1,2	Carboplatin	2	NCT01149083
Veliparib	post-Pt relapsed OC, PPC	BRCA1,2	Topotecan	2	NCT01012817
Veliparib	BC with brain metastases	BRCA1,2	Cisplatin	2	NCT02595905
	TNBC	BRCA1,2	Atezolizuma b +	2	NCT01898117

			Carboplatin/ Cyclophospham ideor Paclitaxel		
Rucaparib	PanC, metastatic digestive system neoplasms	BRCA1,2, PALB2	Fluorouracil, Irinotecan, Leucovorin	1/2	NCT03337087
Rucaparib	CRPC	BRCA1,2, ATM	Docetaxel, Carboplatin	2	NCT03442556
Olaparib	CRPC	HR	Carboplatin	2	NCT04038502
Olaparib	BC	BRCA1,2	Sapacitabine	1	NCT03641755
Rucaparib	FTC,UC,OC (folate receptor alpha positive)	BRCA1,2	Mirvetuxima b (folate receptor)	1	NCT03552471
Olaparib	CRPC	BRCA1,2	Abiraterone Prednisone	2	NCT03012321
Olaparib	OC	BRCA1,2	NA	IIIb	R, №665 / 7339-002-00
Olaparib	OC after Pt	HR	NA	II	R, №594 / 7339-002-00
Talazoparib	BC	BRCA1,2	NA	III	R, №3258 / 673-301
Talazoparib	CRPC	DDR genes	Enzalutamid e (AR)	III	R, №319 / C3441052
Veliparib	HER2- BC	BRCA1,2	Carboplatin Paclitaxel	III	R, №346 / M12-914
Niraparib	hormone-sensitive PC	HR genes	Abiraterone (CYP17A1, AR)	III	R, №16 / 67652000PCR3 00
Niraparib	HER2- BC	BRCA1,2	NA	III	R, №408 / 213831
Rucaparib	recurrent OC, BC, FTC, PPC	BRCA1,2	NA	III	R, №216 / CO-338-043

AR –androgen receptor, BC – breast cancer, CRPC – castration resistant prostate cancer, EndA – endometrial adenocarcinoma, FTC – fallopian tube cancer, HBOC – hereditaory breast and ovarian cancer syndrome HR – homology recombination related deliterious mutation (typically one or more genes: BRCA1, BRCA2, ATM, CDK12, PALB2, ARID1A, ATRX, BLM, BARD1, BRIP1, CHEK1, CHEK2, FANCA, FANCF, FANCG, FANCI, FANCL, FANCM, MSH2, NBN, RAD50, RAD51C, RAD51D, WRN), OC – ovarian cancer, PanC – panceratic cancer, PC – prostate cancer, PPC – primary peritoneal cancer, Pt – platinum based chemotherapy, RTK – receptor tyrosine kinases, R – trials registered in Russia, TNBC – triple negative breast cancer, UCC – uterine corpus carcinoma.
